# A Critical Role for CLSP2 in the Modulation of Antifungal Immune Response in Mosquitoes

**DOI:** 10.1371/journal.ppat.1004931

**Published:** 2015-06-09

**Authors:** Yan-Hong Wang, Yang Hu, Long-Sheng Xing, Hong Jiang, Song-Nian Hu, Alexander S. Raikhel, Zhen Zou

**Affiliations:** 1 State Key Laboratory of Integrated Management of Pest Insects and Rodents, Institute of Zoology, Chinese Academy of Sciences, Beijing, China; 2 Beijing Institute of Genomics, Chinese Academy of Sciences, Beijing, China; 3 Institute of Genetics and Developmental Biology, Chinese Academy of Sciences, Beijing, China; 4 University of Chinese Academy of Sciences, Beijing, China; 5 Department of Entomology and Institute for Integrative Genome Biology, University of California, Riverside, Riverside, California, United States of America; Stanford University, UNITED STATES

## Abstract

Entomopathogenic fungi represent a promising class of bio-insecticides for mosquito control. Thus, detailed knowledge of the molecular mechanisms governing anti-fungal immune response in mosquitoes is essential. In this study, we show that CLSP2 is a modulator of immune responses during anti-fungal infection in the mosquito *Aedes aegypti*. With a fungal infection, the expression of the *CLSP2* gene is elevated. CLSP2 is cleaved upon challenge with *Beauveria bassiana* conidia, and the liberated CLSP2 CTL-type domain binds to fungal cell components and *B*. *bassiana* conidia. Furthermore, *CLPS2* RNA interference silencing significantly increases the resistance to the fungal challenge. RNA-sequencing transcriptome analysis showed that the majority of immune genes were highly upregulated in the CLSP2-depleted mosquitoes infected with the fungus. The up-regulated immune gene cohorts belong to melanization and Toll pathways, but not to the IMD or JAK-STAT. A thioester-containing protein (TEP22), a member of α_2_-macroglobulin family, has been implicated in the CLSP2-modulated mosquito antifungal defense. Our study has contributed to a greater understanding of immune-modulating mechanisms in mosquitoes.

## Author Summary

Entomopathogenic fungi represent a promising class of bio-insecticides for mosquito control. Detailed knowledge of molecular mechanisms governing anti-fungal immune response in mosquitoes is essential. CLSP2 composed of serine protease and lectin domains functions as a modulator of the mosquito immune system during the anti-fungal response. Transcriptome analysis indicated that the Toll pathway and melanization genes are highly up-regulated in CLSP2 RNA interference depleted mosquitoes infected with the fungus *Beauveria bassiana*. A thioester-containing protein TEP22, a member of α_2_-macroglobulin family, is involved in the CLSP2-modulated mosquito antifungal defense. Our study has contributed to the understanding of immune-modulating mechanisms in mosquitoes.

## Introduction

Female mosquitoes require repeated blood feedings during their life cycle to satisfy their reproductive nutritional needs and, as a consequence, they serve as vectors of numerous human diseases [1]. Malaria, transmitted by the *Anopheles* genus, is the most devastating vector-borne human disease and causes about one million deaths per year. The annual number of cases of Dengue fever, a viral disease transmitted by *Aedes aegypti*, has reached over a hundred million. Major reasons for this serious situation include the lack of effective vaccines against major mosquito-borne diseases, rapidly developing drug and insecticide resistance, and socio-economic problems in endemic countries.

It is imperative to design novel specific biological pesticides, since mosquitoes have developed resistance to most currently used chemical insecticides [2]. While entomopathogenic fungi *Beauveria bassiana* and *Metarhizium anisophliae* infect insects by direct penetration of the cuticle, bacteria and viruses often need to be ingested, which makes fungi more promising as pesticides. However, fungal pathogens still require improvements due to the relatively low virulence when compared with chemical pesticides [3]. Detailed studies of antifungal immunity in mosquitoes are essential for future improvements of fungal biocontrol agents.

Multicellular organisms have evolved complex and powerful systems of immune responses to counteract continuous attacks of various pathogens. An essential feature of the immune system in any organism is its capacity to sustain equilibrium between reactivity and quiescence [4]. A loss of such a balance leads to severe consequences, such as autoimmune and inflammatory diseases in humans. Inhibitory receptor systems modulating immune responses have been identified in vertebrates [4,5]. However, the detail mechanism of the analogous system in insect is still not very clear. Our studies have revealed that CLSP2 functions as a key modulator of the mosquito immune system and contributed to a better understanding of immune modulating mechanisms in insects.

In insects, Toll is the principal innate immune pathway responsible for the anti-fungal response [6,7,8]. The Toll pathway is induced by fungal β1,3-glucan and also by Gram-positive bacteria harboring Lys-type peptidoglycan [7]. This pathway is crucial in activating immune responses especially in production of antimicrobial and anti-fungal peptides (AMPs) [6,9,10]. Gram-negative binding protein 3 (GNBP3), a member of the β-1, 3-glucan recognition protein (βGRP) family, binds to fungal cell components and initiates the Toll pathway [11]. Two Clip domain serine proteases (CLIPs)—Persephone and Späetzle-processing enzyme (SPE)—are components of an extracellular serine protease (SP) cascade and cause the cleavage of a cysteine knot cytokine, Späetzle (Spz) [12,13]. The cleaved Spz then functions as a ligand of the Toll receptor, which in turn passes the signals into the intracellular signal cascade consisting of MyD88, Tube, Pelle and TRAF6. Mosquitoes have a single orthologue of Dorsal, Rel1 [6]. Activation of the intracellular signal cascade by Toll results in the phosphorylation and degradation of Cactus, which is an inhibitor of the NF-кB transcription factors Dorsal and Dif [14]. Removal of Cactus releases and causes nuclear translocation of Dorsal and Dif, which eventually leads to the expression of AMPs, including Drosomycin, an antifungal peptide. Previously, *Ae*. *aegypti* orthologues of *Drosophila* genes of the Toll pathway—Spz1C, Toll5A, CLIPB5, and CLIPB29—have been identified and shown to mediate the Toll pathway in response to fungal infection [6,15]. In mosquitoes, Cecropins and Defensins are the major AMPs involved in the systemic antifungal immune responses [16].

Melanization represents a second immune pathway that is essential in the systemic antifungal immune responses [17]. It is the arthropod-specific defense mechanism that plays an essential role in wound healing and innate immunity [17]. The key enzymes for this reaction are prophenoloxidases (PPOs), which, once activated, catalyze the formation of toxic melanin. Melanin is then deposited around the wound or invading pathogens, including fungi. CLIPs constitute a cascade for amplification of a signal triggered by pathogen infection that results in PPO cleavage into an active PO by a melanization protease (MP). The melanization cascade is tightly regulated by serine protease inhibitors (SRPNs), which prevent spontaneous initiation of the reaction. The analysis of the mosquito genomes has shown that genes encoding immune signaling and effector molecules, and the number of melanization pathway genes have undergone major expansion [16]. For example, there are 10 PPO genes in the *Ae*. *aegypti* genome [13]. However, the precise roles of each PPO in melanization process are poorly understood. Our previous study revealed a novel level of complexity in the melanization cascade of the mosquito *Ae*. *aegypti*. Namely, we identified that there are several independent pathways leading to melanization, each requiring a different protease/SRPN regulatory module [15]. Of particular interest is a clear separation of tissue melanization, represented by melanin tumors often associated with the damage of host tissues, and immune melanization involved in the recognition and killing of pathogens, including fungi [13]. The melanization response has also been shown to significantly retard the growth and dissemination of *B*. *bassiana* in the *An*. *gambiae* mosquito [18].

Previously, we have identified an immune factor in *Ae*. *aegypti*, CLSP2 (AAEL011616), that is composed of an elastase-like serine protease (ESP) and CTL-type domains [19]. In this study, we show that CLSP2 is the key negative modulator of immune responses during anti-fungal infection. The expression of the *CLSP2* gene is elevated upon *B*. *bassiana* infection. CLSP2 is cleaved upon challenge with *B*. *bassiana* and the liberated CLSP2 CTL-type domain binds to fungal cell components. Moreover, RNAi depletion of CLPS2 (iCLSP2) significantly increases the resistance to the fungal challenge. RNA-sequencing (RNA-seq)-based transcriptome analysis indicated that the Toll pathway and melanization genes are highly up-regulated in CLSP2 RNAi-silenced mosquitoes infected with *B*. *bassiana* (iCLSP2Bb). TEP22, a member of α_2_-macroglobulin family, was identified to be regulated by CLSP2 and to participate in the antifungal immune response in the *Ae*. *aegypti* mosquitoes.

## Results

### CLSP2 responses to fungal infection

We investigated the CLSP2 responses to fungal infection at the gene and protein levels. Real-time RT-PCR (qPCR) analysis showed that the CLSP2 mRNA level in mosquitoes was significantly up-regulated after septic injections of conidia of the fungus *B*. *bassiana* (S1A Fig). This result was consistent with our previously reported Northern results [19], indicating a CLSP2 response to infections at the gene level.

**Fig 1:**
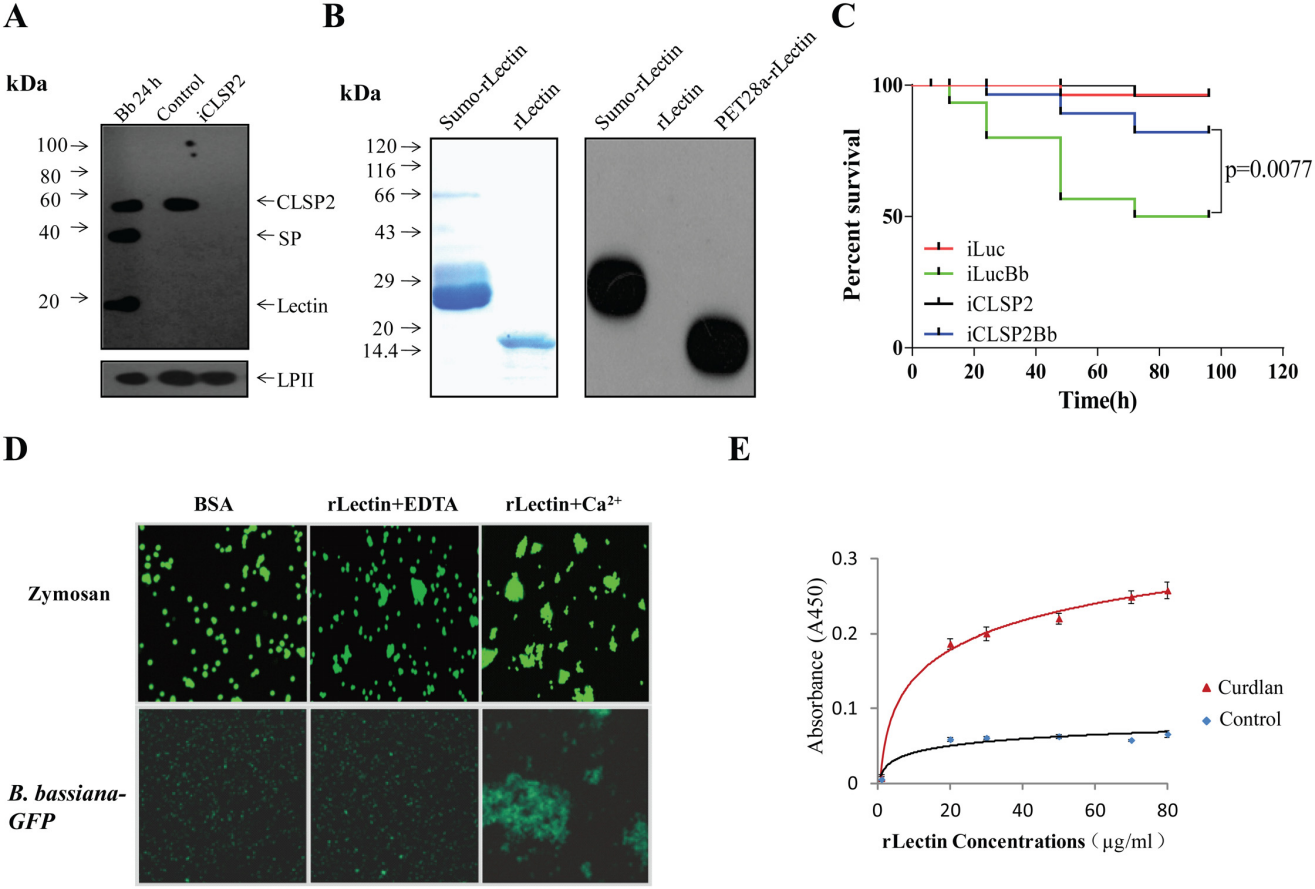
CLSP2 responses to infection by the fungus *B*. *bassiana* and isolation of rLectin. **A)** Immunoblot analysis showing the composition and cleavage of CLSP2 in iCLSP2, and mosquitoes after fungal challenge. Hemolymph was isolated from mosquitoes 24 h after treatment with *B*. *bassiana* (Bb 24h). Control, mosquitoes were treated with (PBS). iCLSP2, CLSP2 RNAi depleted mosquitoes injected with PBS. The 14-kDa lectin domain and the 33-kDa SP domain bands were present in hemolymph samples from Bb 24h mosquitoes, but not those from Naïve ones. CLSP2 polyclonal antibodies were used in the immunoblot analysis; *Ae*. *aegypti* Lipophorin II was used as the loading control. **B**) Purified sumo-rLectin and rLectin analyzed on 10% SDS-PAGE. To generate rLectin, the sumo-rLectin was cleaved by SUMO protease, and sumo tag was removed using a nickel affinity column. SDS-PAGE with Coomassie blue staining of Sumo-rLectin and rLectin (left panel). Western blot analysis of purified sumo-rLectin, rLectin, and PET28a-rLectin, which is cloned in PET28a and contains His-tag (right panel). Sumo-rLectin and PET28a-rLectin were detected using the anti-Histidine monoclonal antibody. **C**) Survival rate of mosquitoes infected with *B*. *bassiana* conidia was partially rescued by CLSP2 RNAi depletion (iCLSP2Bb). The survival rate of iLucBb mosquitoes was significantly different from that of the iLuc and iCLSP2Bb mosquitoes (p < 0.01). Each experiment was performed in three replicates. iLucBb, iLuc-depleted mosquitoes infected with *B*. *bassiana*; iCLSP2Bb, CLSP2 RNAi depleted mosquitoes infected with *B*. *bassiana*; iCLSP2, CLSP2 RNAi depleted mosquitoes injected with PBS; iLuc, luciferase RNAi-treated control mosquitoes injected with sterile phosphate buffered saline (PBS). See Materials and Methods for details. **D**) The agglutination assay testing interaction of rLectin with zymosan and GFP-conjugated *B*. *bassiana*. Purified rLectin samples (Fig 1B) were incubated with FITC-labeled zymosan or *B*. *bassiana*-GFP in addition of either 0.5 mM EDTA (rLectin + EDTA) or 5 mM CaCl_2_ (rLectin + Ca^2+^) for 45 min. The left panel is a control in which zymosan and *B*. *bassiana*-GFP were incubated with 1 mg/ml BSA. **E**) ELISA test of rLectin binding to the fungal cell wall component, curdlan. rLectin was prepared at different concentrations (0–80 μg/ml) in binding buffer containing 5 mM CaCl_2_ and BSA for 20 min. rLectin was added to curdlan-coated microtiter plates, and the binding assay was performed as described in Materials and Methods. The control was tested by pre-immune antiserum. Each point represents the mean of four individual measurements ± SEM.

*Aedes* CLSP2 consists of two domains: the N-terminal elastase-like serine protein (ESP) and the C-terminal galactose-type C-type lectin (CTL), which includes a signature QPD sequence. CLSP2 includes a signal peptide and no transmembrane domain, suggesting that it is a secreted peptide. To investigate the infection effect on the CLSP2 protein composition, hemolymph samples were resolved on SDS-PAGE and subjected to immunoblot analysis utilizing anti-CLSP2 polyclonal antibodies. In the hemolymph of control mosquitoes injected with sterile phosphate buffered saline (PBS), CLSP2 remained as a single band of about 47 kDa, which disappeared in RNA-interference (RNAi) CLSP2 silenced mosquitoes (iCLSP2) (Fig 1A). However, two bands, corresponding to the 33-kDa ESP and 14-kDa lectin domains, were detected in the mosquito hemolymph after infection with *B*. *bassiana* conidia (Fig 1A). This suggested that CLSP2 was cleaved upon immune challenge. When we used the hemolymph from mosquitoes with silenced CLSP2, no bands were evident in the immunoblot, indicating that the 33-kDa and 14-kDa bands observed in the mosquito hemolymph after infection with *B*. *bassiana* conidia belong to CLSP2. Additional controls for the specificity of anti-CLSP2 antibodies are presented in S1B and S1C Fig.

In an attempt to understand the biochemical properties of CLSP2, we cloned and produced its lectin domain, designated as rLectin, using an *Escherichia coli* expression system. Myc tag rLectin fused with hexahistidine-tagged SUMO was purified using an affinity Ni-NTA agarose column. The isolated product was cleaved by SUMO protease, and then reloaded onto the Ni-NTA column, so that rLectin was in the flow-through fraction and the His-tagged protease was retained on the column. The purified rLectin migrated as a single band with the expected molecular weight (MW) of 14 kDa on SDS-PAGE (Fig 1B) that was not recognized by the anti-Histidine monoclonal antibody (Fig 1B).

To address the CLSP2 role in immune responses, we investigated the susceptibility of iCLSP2 mosquitoes to fungal infections. Three days after CLSP2 dsRNA injection, mosquitoes were infected with *B*. *bassiana* and their survival rate was evaluated. The survival rate of iCLSP2 mosquitoes challenged with *B*. *bassiana* (iCLSP2Bb) was significantly higher than that of mosquitoes infected with this fungus alone in the iLuc background (iLucBb). Mosquitoes with Luciferase (Luc) gene silencing served as a control (iLuc) (Fig 1C). qPCR and immunoblotting tests confirmed efficiency of CLSP2 RNAi (S2A–S2C) Fig. Thus, silencing of CLSP2 in mosquitoes led to an increased resistance to fungal infection, suggesting a role of CLSP2 in modulating immune activation.

### The CLSP2 lectin domain is responsible for fungal recognition

In order to decipher the interaction between CLSP2 and fungi, we examined binding properties of rLectin by means of the agglutination assay. As fungal representatives, we tested zymosan, which is a component of the *Saccharomyces cerevisiae* cell wall composed of β-glucans and mannan, and GFP-conjugated *B*. *bassiana* in the rLectin agglutination assay (Fig 1D). Neither zymosan nor GFP-conjugated *B*. *bassiana* aggregates were observed in the presence of bovine serum albumin (BSA) used as a control. Only minor aggregates were found in the presence of EDTA. However, large aggregates were observed in the presence of Ca^2+^, indicating that it was required for the agglutination reaction by rLectin of either zymosan or GFP-conjugated *B*. *bassiana* (Fig 1D).

Next, we performed enzyme-linked immunosorbent assay (ELISA) to test whether rLectin directly bound to the fungal cell component curdlan. Different amounts of rLectin (20, 30, 50, 70 and 80 μg/ml; 50 μl each) were added to microtiter plate wells coated with curdlan, and the bound rLectin was detected using Myc antibodies. ELISA has demonstrated that rLectin effectively binds to curdlan (Fig 1E). Taken together, these results suggest that the CLSP2 lectin domain is capable of recognizing and binding to fungal carbohydrate surface molecules in a saturable and Ca^2+^ dependent manner.

### CLSP2 modulates expression of a large array of genes involved in innate immunity

**Fig 2:**
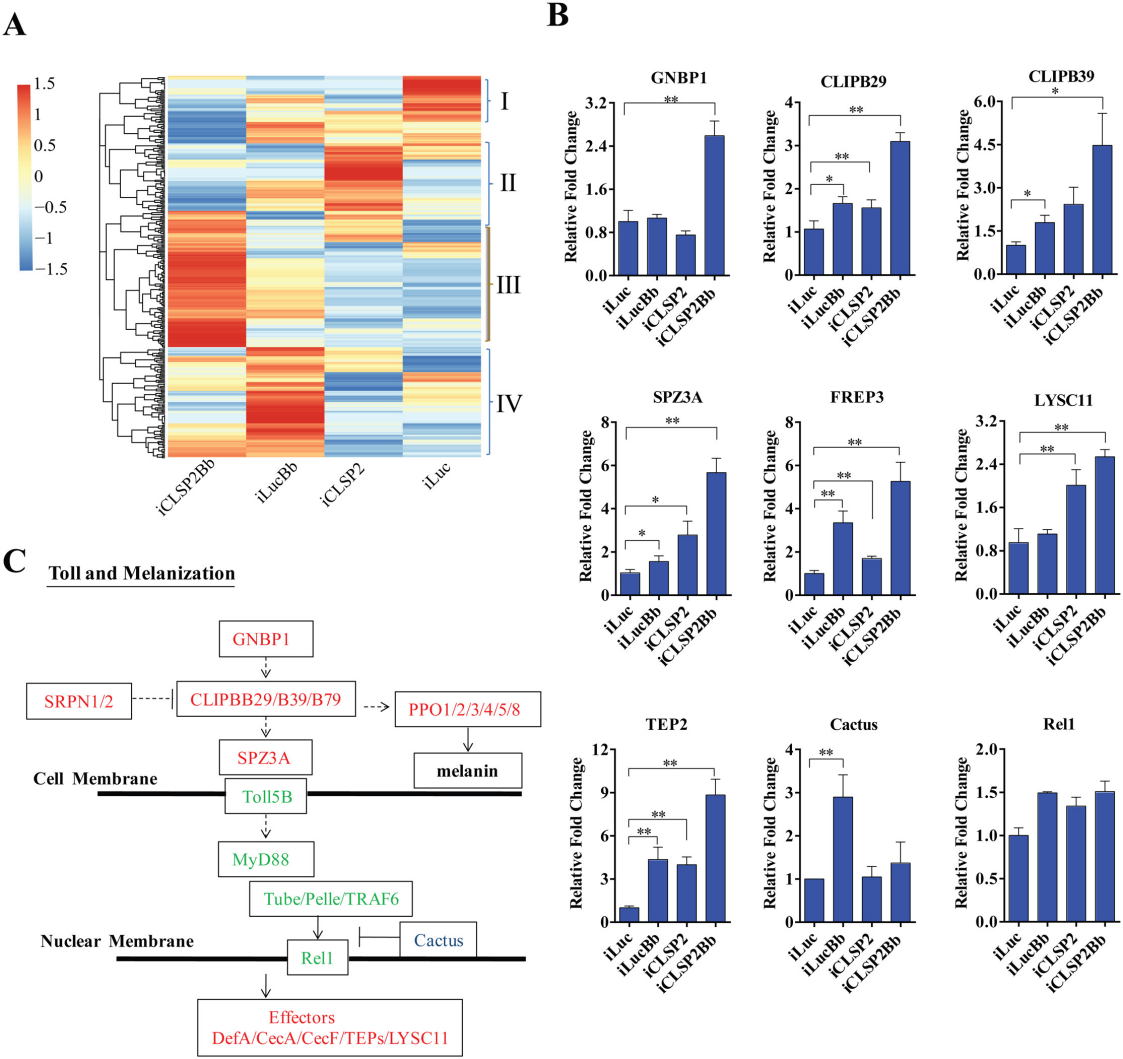
Transcriptome analysis of CLSP2 modulation of *Ae*. *aegypti* immune genes. **A**) Hierarchical cluster analysis of gene transcripts in mosquito tissue (abdominal carcasses) after four different treatments: iLuc, iCLSP2, iCLSP2Bb, and iLucBb (left panel). Clusters I-IV represent gene cohorts that are specifically up-regulated in iLuc (Cluster I), iCLSP2 (Cluster II), iCLSP2Bb (Cluster III) and iLucBb (Cluster IV). Hierarchical cluster analysis of Cluster III immunity-related genes up-regulated in iCLSP2Bb mosquitoes is shown in S2 Fig. Transcript abundance of each gene was calculated using the RSEM software package (Trinity) and heat maps generated by R software. The color scale indicates the abundance deviation from the median for each gene. The list of up-regulated genes in each group was shown in S1–S3 Tables. **B**) Real-time RT-PCR validation of transcript levels of selected immune genes. Data were normalized to the expression level of iLuc. Data were shown as mean ± SEM. * p < 0.05; ** p < 0.01. iLucBb, iLuc mosquitoes infected with *B*. *bassiana* (24 h post infection); iCLSP2, CLSP2 dsRNA-treated mosquitoes infected with *B*. *bassiana* (24 h post infection); iCLSP2, mosquitoes injected with CLSP2 dsRNA. Control group (iLuc) was injected with luciferase dsRNA. **C**) A schematic diagram of the Toll and melanization pathways. Red represents genes up-regulated in iCLSP2Bb (ratios ≥ 1.5 fold), while those that were not significantly affected are shown in green. Cactus, which inhibited in iCLSP2Bb, is in blue.

Next, CLSP2 effect on expression of immune genes was elucidated by means of the RNA-sequence-based transcriptome analysis (RNA-seq) linked with RNAi screens. For this analysis, we used mosquitoes after three different treatments: control iLuc infected with *B*. *bassiana* (iLucBb), silenced with CLSP2 RNAi (iCLSP2), silenced with CLSP2 RNAi and infected *B*. *bassiana* (iCLSP2Bb). iLuc mosquitoes served as a control. Regulated immune gene repertoire (fold change ≥ 1.5) in different groups is shown in S1–S3 Tables. The hierarchical clustering analysis has revealed a stunning up-regulation of major immune gene transcripts in iCLSP2Bb mosquitoes (Fig 2A and S2D Fig).

Serine proteases (SPs) play important roles in a wide range of biological processes, including innate immunity. They constitute an integral part of immune reactions, such as the Toll and melanization cascades in arthropods [7,14]. There were 40 CLIPs (almost half of *Ae*. *aegypti* genome CLIPs) in the iCLSP2Bb upregulated transcriptome (S3 Table). A high elevation of expression levels of several immune genes in iCLSP2Bb mosquitoes was confirmed by means of the qPCR analysis (Fig 2B and S2E Fig). Our previous study has shown that CLIPB5 and CLIPB29 are involved in the activation of Toll pathway by fungal infection or by infection-independent manner, respectively [15]. Indeed, we found that both CLIPB5 and CLIPB29 were moderately up-regulated in iCLSP2Bb (S3 Table). Two Toll pathway regulators—Spz2 and Spz3A—were also dramatically up-regulated in the iCLSP2Bb mosquitoes (Fig 2A and 2B and S2E Fig). Moreover, the gene encoding the pattern recognition receptor GNBP1 was also significantly activated in iCLSP2Bb mosquitoes. Thus, our results have shown that CLSP2 modulates the transcriptional expression of the Toll pathway upstream genes (Fig 2C). However, the expression of genes encoding intracellular components of the intracellular Toll pathway signaling, including Rel1, was not significantly affected in these mosquitoes (Fig 2B and 2C). Interestingly, *Cactus* was elevated as a result of *B*. *bassiana* infection, however its transcript was reduced in iCLSP2Bb (Fig 2B).

Genes encoding pattern recognition receptors from the fibrinogen-related protein family (FREP) represented another highly elevated group of genes in the iCLSP2Bb mosquitoes (Fig 2B and S2E Fig, S3 and S4 Tables). *FREP3*, *FREP5* and *FREP10* were particularly up-regulated. The FREPs are an evolutionarily conserved immune gene family found in mammals and invertebrates [20]. It is the largest pattern recognition receptor gene family in mosquitoes, with 59 putative members in *An*. *gambiae* [20] and 35 in *Ae*. *aegypti* [16]. Genes encoding thioester-containing proteins, TEP2, TEP3 and TEP22 were also up-regulated. These data suggest that CLSP2 is an immune factor working upstream of the pattern-recognition receptor system, modulating their responses.

**Fig 3:**
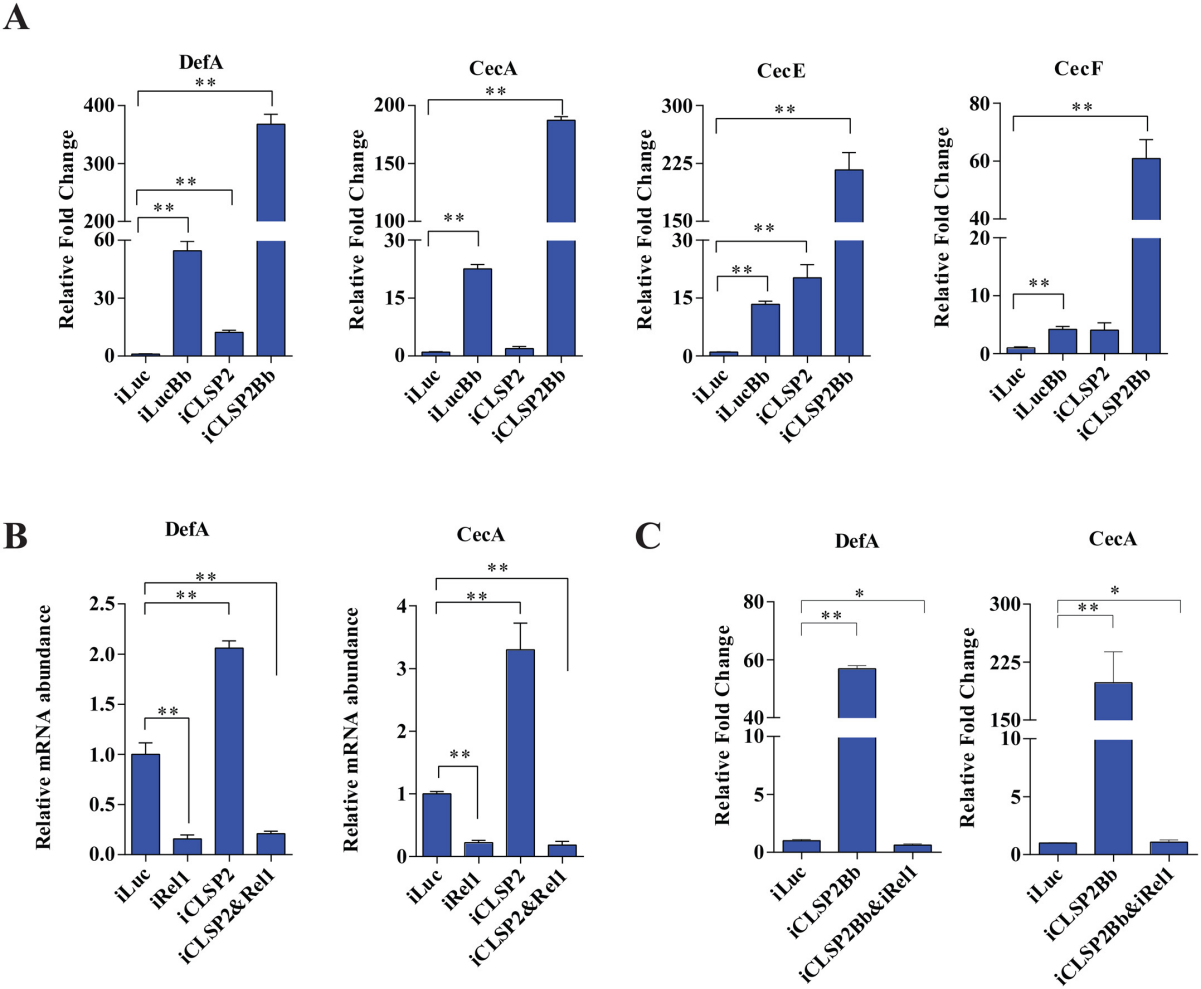
The effect of CLSP2 on the expression of AMPs. **A)** The mRNA levels of *DefA*, *CecA*, *CecE*, and *CecF* were determined using real-time RT-PCR. Data are presented as mean ± SEM. *, p < 0.05; **, p < 0.01. The experiments were repeated four times. Data were normalized to the expression level of iLuc. iLucBb, iLuc-depleted mosquitoes infected with *B*. *bassiana*; iCLSP2Bb, CLSP2 RNAi depleted mosquitoes infected with *B*. *bassiana*; iCLSP2, CLSP2 RNAi depleted mosquitoes; iLuc, luciferase RNAi-treated control mosquitoes. **B-C)** The effect of double-knockdown of CLSP2 and Rel1 on the expression of AMPs (B) and the response to fungal challenge (C). The mRNA levels of *DefA*, *CecA* were determined using qRT-PCR. Data are presented as mean ± SEM. **, p < 0.01. Data were normalized to the expression level of iLuc. iCLSP2, mosquitoes treated with CLSP2 dsRNA; iRel1, mosquitoes treated with Rel1 dsRNA; iCLSP2&Rel1, mosquitoes treated with CLSP2 and Rel1 dsRNA; iLuc, luciferase RNAi-treated control mosquitoes. iCLSP2Bb&Rel1 and iCLSP2Bb, CLSP2 and Rel1 RNAi depleted mosquitoes infected with *B*. *bassiana*.

We then studied the effect of CLSP2 on mRNA levels of anti-microbial effector peptides (AMPs). Defensin A (DefA) and Cecropin A (CecA) represent the major mosquito AMPs [16], which also convey anti-*Plasmodium* activity [21]. As shown using qPCR, the mRNA levels of AMP genes, *DefA*, *CecA*, *CecE*, and *CecF* were induced in *B*. *bassiana* infected mosquitoes at 20- to 40-fold levels. Impressively, much higher induction levels of *DefA*, *CecA*, *CecE*, and *CecF* were observed in the iCLSP2Bb mosquitoes (Fig 3A). Thus, the CLSP2 played essential role in systemic immunity in mosquitoes by preventing the spontaneous transcription activation of downstream AMP immune genes. Next, we investigated interrelationship of CLSP2 and Rel1, which is a factor directly controlling the expression of AMP genes. As expected, the high expression of *DefA* and *CecA* brought by iCLSP2 was significantly decreased in mosquitoes with double CLSP2 and Rel1 RNAi silencing (Fig 3B). Moreover, the extremely high level of AMP expression in iCLSP2Bb mosquitoes was almost completely eliminated in mosquitoes with double knockdown of CLPS2Bb and Rel1 (Fig 3C). These experiments indicate that the action of CLSP2 due to modulation of upstream regulatory factors of the Toll immune cascade. According to the result from our RNA-seq analysis and qPCR, the induced immune genes belong mainly to the Toll pathway (Fig 2C), whereas the genes involved in the IMD and JAK/STAT pathways were not affected by the depletion of CLSP2 (S3A Fig). The only exception is PGRP-LC, which was influenced by CLSP2 and surprisingly by fungal challenge (S3B Fig). However, unlike GNBP1, it was not up-regulated the in iCLSP2Bb mosquitoes, thus pointing to the lack of modulation of this gene encoding the IMD pattern-recognition receptor by CLSP2. Moreover, CLSP2 had no effect on expression of the *Rel2* gene, the principal regulator of the IMD pathway (S3A and S3B Fig). Toll and IMD share regulation of AMPs [22]. However, our experiments strongly suggest that CLSP2 effect on expression of the *AMP* genes is likely solely due to its influence on the Toll pathway.

**Fig 4:**
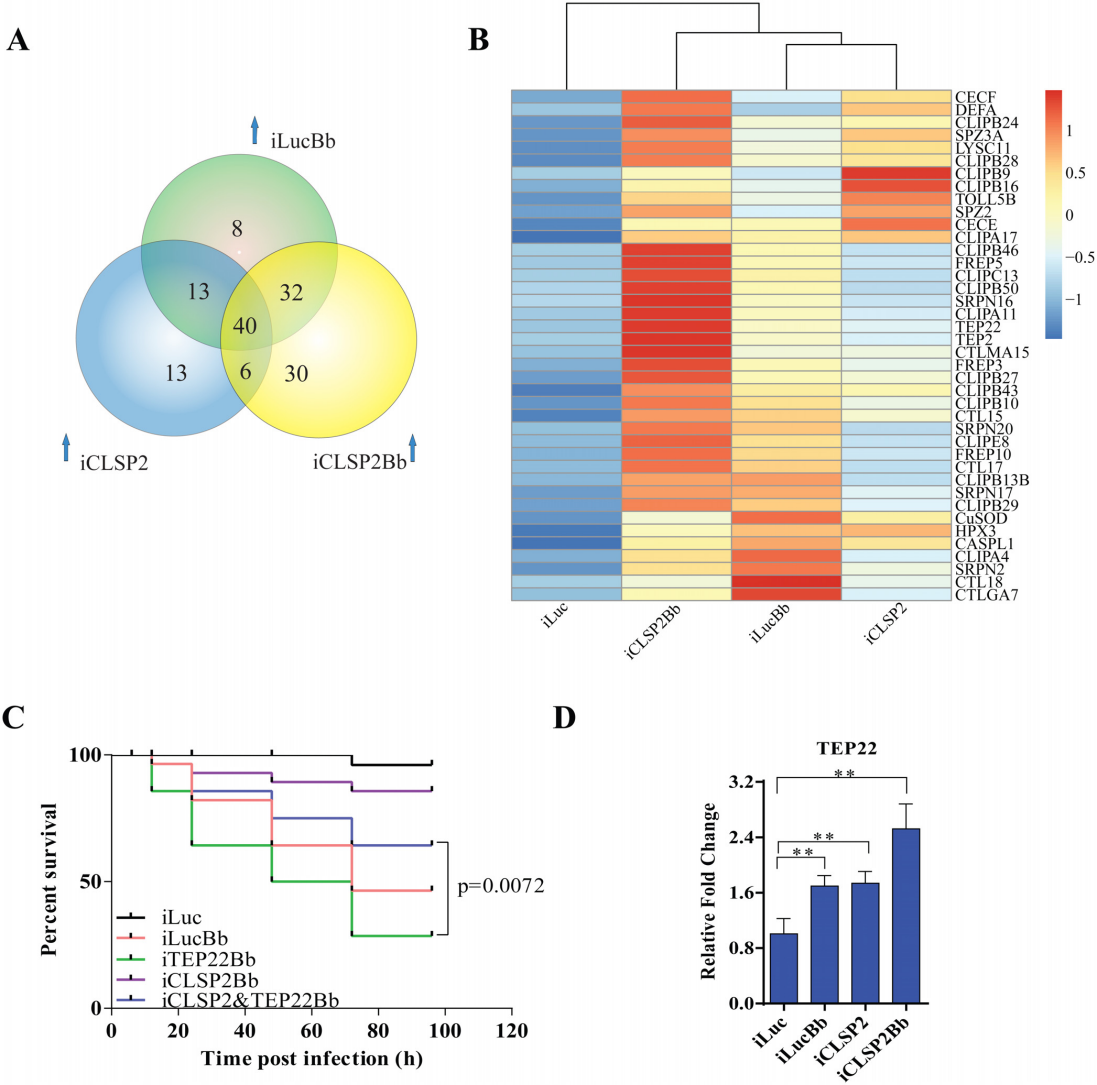
Comparative transcriptome analysis of CLSP2 modulation of immune genes and the role of TEP22 in anti-fungal defense. **A**) Venn diagram representing unique and shared up-regulated immune genes (1.5 fold changes compared with control) in iLucBb, iCLSP2, and iCLSP2Bb mosquitoes. **B**) Expression profiles of immune genes that are concurrently up-regulated in iLucBb, iCLSP2, and iCLSP2Bb mosquitoes. Transcript abundance of each gene was calculated using the RSEM software package (Trinity) and heat maps generated by R software. The color scale indicates the abundance deviation from the median for each gene. Expression levels of common up-regulated genes are also shown in S4 Table. **C**) The survival rate of mosquitoes showed that concomitant depletion of *TEP22* and *CLSP2* (iCLSP2&iTEP22Bb) enhanced the capacity of mosquitoes to defend *B*. *bassiana* (p < 0.01) compared with single depletions of iTEP22Bb, or control iLucBb. Each experiment was performed in three replicates. Green line—iTEP22Bb, TEP22 dsRNA-treated mosquitoes infected with *B*. *bassiana*; purple line—double knockdown iCLSP2&TEP22 mosquitoes infected with *B*. *bassiana*; blue line—iCLSP2 mosquitoes injected with *B*. *bassiana* conidia; red line—iLucBb, iLuc mosquitoes injected with the fungus; black line—iLuc control mosquitoes. **D**) mRNA abundance of *TEP22* was up-regulated in iCLSP2Bb mosquitoes. Normalized expression level of iLuc was shown. Data were shown as mean ± SEM. * p < 0.05; ** p < 0.01. iLucBb, iLuc mosquitoes infected with *B*. *bassiana* (24 h post infection); iCLSP2, CLSP2 dsRNA-treated mosquitoes infected with *B*. *bassiana* (24 h post infection); iCLSP2, mosquitoes injected with CLSP2 dsRNA. Control group (iLuc) was injected with luciferase dsRNA.

We next selected up-regulated gene cohorts from mosquitoes after three different treatments—iCLSP2 (72 genes), iLucBb (93 genes) and iCLSP2Bb (108 genes) for further analysis. Forty immune genes were induced under all three experimental conditions (Fig 4A and S4 Table). The ontology analysis demonstrated that, except for several effector genes, the majority of co-upregulated genes belonged to regulatory categories located upstream of immune cascades (Fig 4B and S4 Table). The hierarchical clustering indicated that transcript levels of most of these genes are considerably higher in iCLSP2Bb than in the other two groups (Fig 4B). These results further suggest that CLSP2 is the modulator of the immune response involved in the anti-fungal infection.

### Thioester-containing protein (TEP22) is involved in the CLSP2-modulated mosquito antifungal defense

Our analysis suggests that the modulating factor CLSP2 acts upstream of immune cascades, possibly interacting with other factors. To explore this possibility, we analyzed eight genes selected from those co-up-regulated in iLucBb, iCLSP2 and iCLSP2Bb mosquitoes (S5 Table), and their functions were studied by means of RNAi depletions in a combination with *B*. *bassiana* infection. After treatment with TEP22 dsRNA, a member of α_2_-macroglobulin family (S4 Fig), mosquitoes became extremely sensitive to the *B*. *bassiana* infection, and the survival rate dramatically decreased. However, the survival of affected mosquitoes could be partially rescued after the knockdown of *CLSP2* was performed simultaneously with that of *TEP22* (Fig 4C). Additionally, TEP22 was significantly regulated in iCLSP2Bb mosquitoes (Fig 4D). The results indicate that TEP22 is required for the anti-fungal response in a mosquito. Moreover, these results suggested that CLSP2 is likely mediated the response to fungal infection via interaction with TEP22 as a recognition molecule. Seven other tested genes from the iCLSP2Bb did not yield a similar phenotype indicating that they were not involved in the CLSP2 immune modulation directly (S5 Fig).

### CLSP2 differentially modulates the transcription of *PPO* genes

**Fig 5:**
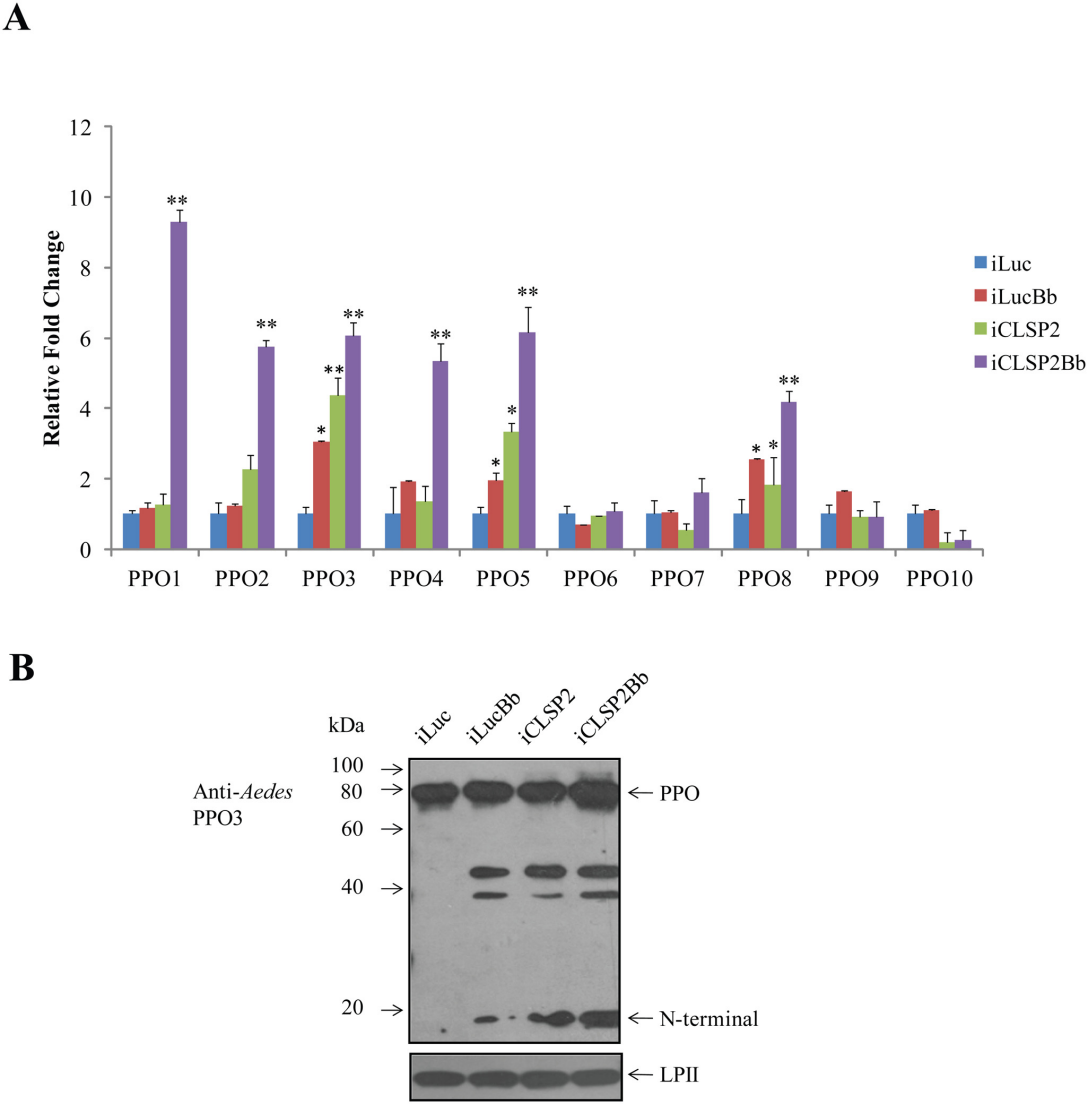
The effect of CLSP2 on transcriptional activation of *PPO* genes. **A**) The effect of CLSP2 on the expression of 10 *PPO* genes in the mosquito carcass. The mRNA levels of *PPOs* were determined using real-time RT-PCR. Data were represented as mean ± SEM. *, p < 0.05; **, p < 0.01. The experiments were repeated four times. iLucBb, iLuc mosquitoes injected with *B*. *bassiana*; iCLSP2Bb, CLSP2 dsRNA-treated mosquitoes injected with *B*. *bassiana*; iCLSP2, mosquitoes injected with CLSP2 dsRNA; iLuc, luciferase RNAi-treated control mosquitoes. **B**) Immunoblot analysis of hemolymph PPO cleavage. Hemolymph was isolated at 24 h post-infection from four groups (iLuc, iLucBb, iCLSP2, and iCLSP2Bb, see Material and Methods) of mosquitoes, separated by SDS-PAGE, and then probed with *Ae*. *aegypti* PPO3 polyclonal antibodies. A ~20-kDa band appeared in samples of iLucBb, iCLSP2 and iCLSP2Bb. *Ae*. *aegypti* Lipophorin II was used as the loading control. iLucBb, iLuc mosquitoes infected with *B*. *bassiana*; iCLSP2Bb, CLSP2 dsRNA-treated mosquitoes infected with *B*. *bassiana*; iCLSP2, mosquitoes treated with CLSP2 dsRNA; iLuc, luciferase dsRNA treated control mosquitoes.

CLSP2 has been shown to be a negative modulator of hemolymph melanization [19]. To examine whether CLSP2 was involved in regulation of *PPO* gene expression, we utilized CLSP2 RNAi silencing in combination with *B*. *bassiana* infection (Fig 5A). The RNA abundance of 10 *Aedes PPOs* was investigated by means of qPCR analysis. Whereas transcript abundance of *PPO* genes did not change significantly after infection with *B*. *bassiana* alone, a highly pronounced activation of several *PPO* genes was observed in the iCLSP2Bb mosquitoes. *PPO1* transcript increased dramatically by 9-fold, while levels of PPO2, PPO3, PPO4, PPO5 and PPO8 were elevated to about 4- to 6- fold. These results suggest that CLSP2 is an essential modulator of PPO gene expression. Moreover, this modulation is highly specific to just a few PPO genes that are most likely involved in immune responses during fungal infection. In addition to PPO genes, according to the results of RNA-seq, we also found that transcripts of several genes involved in melanization were up-regulated in iCLSP2Bb mosquitoes, including *CLIPB9* and *SRPN2* (S3 Table). The elevation of these gene transcripts in iCLSP2Bb was confirmed by qPCR (S2E Fig).

We selected PPO3 for further protein analysis, because its gene transcript was elevated in response to the fungal infection in iLucBb and was also highly upregulated in the iCLSP2Bb mosquitoes. Proteolytic cleavage of hemolymph PPO3 was detected by immunoblotting using polyclonal antibodies against *Aedes* PPO3. There was only a precursor PPO band in the iLuc control mosquitoes (Fig 5B). However, it was cleaved in the hemolymph of *B*. *bassiana-*infected and CLSP2-silenced mosquitoes as marked by the appearance of around 20-kDa-protein band (Fig 5B). The PPO3-derived cleavage proteins were also observed in iCLSP2Bb mosquitoes. At present we cannot assume that CLSP2 is responsible for cleavage of only PPO3 as we lack specific antibodies to other PPOs to investigate this question. The cleavage of PPO is likely not a direct effect of CPLS2 and occurs as a consequence of activation of melanization pathway factors by iCPLS2 and/or infection with *B*. *bassiana* as shown above. However, this experiment demonstrates importance of CLSP2 as a modulating factor working upstream in the PPO cascade.

## Discussion

Innate immune responses are initiated by the interaction between pathogen surface molecules and pathogen-related receptors (PRRs). C-type lectin recognition receptors (CTL or CTR) comprise a large family of PRRs that are engaged in the recognition of a broad spectrum of pathogens. They are also defined as Ca^2+^—dependent carbohydrate (lectin) binding proteins identified in a wide range of animal groups [23]. CTLs interact with glycans on cell surfaces, in the extracellular matrix, or on soluble secreted glycoproteins, mediating processes such as cell adhesion, cell-cell interactions and pathogen recognition [23].

CTLs have been implicated in pathogen evasion of a host immune system. In mammals, a large family of C-type lectin receptors modulating immune responses has been characterized[24]. Two CTLs (CTL4 and CTLMA2) have been shown to act as protective agonists during the development of *Plasmodium* ookinetes to oocysts in the mosquito *An*. *gambiae* [25]. Similarly, another C-type lectin, mosGCTL-1, an equivalent to CTLMA15, was found to facilitate the infection of West Nile virus in the mosquito *Ae*. *aegypti* [26]. Although CTLs have been identified as negative regulators of the immune response against malaria parasites and virus [25,26], details of mosquito CTL-based pathways are still unknown. Mosquitoes with the CLSP2 RNAi depletion displayed an elevated resistance to *B*. *bassiana* infection as compared to those with pathogens alone. Previously, we have also demonstrated that CLSP2 also functions during *Plasmodium* infection [17]. Thus, our study has uncovered an important role of CLSP2 as a factor modulating immune responses in the mosquito *Ae*. *aegypti*.

*Ae*. *aegypti* CLSP2 is a composite protein consisting of an elastase-like SP (ESP) domain located at the N-terminal and a CTL-type domain at the C-terminal. Composite immune proteases, such as *Manduca sexta* HP14 and the factor C of the horseshoe crab *Tachypleus tridentatus*, undergo cleavage after immune challenge [27,28]. Our experiments have shown that CLSP2 is also cleaved upon challenge with *B*. *bassiana*. Two bands were detected corresponding to the 14-kDa lectin and 33-kDa ESP domains in the hemolymph samples of mosquitoes with fungal infection by means of immunoblot analysis utilizing anti-CLSP2 antibodies. We have provided two lines of evidence clearly showing that the CLSP2 CTL-type domain binds to fungal sugar cell components. The purified recombinant CTL-type domain (rLectin) agglutinates zymosan and *B*. *bassiana* conidia in a calcium-dependent manner. Moreover, ELISA has shown that rLectin directly binds to the polysaccharide fungal cell component, curdlan, and this binding is saturable. However, whether the CTL domain binding to fungal surface molecules occurred before or after the CLSP2 cleavage in the hemolymph could not be determined. It also remains to be clarified whether upon infection-induced cleavage the CLSP2 domains undergo conformational changes still remaining as a single molecule in its native state or yielding completely separate molecules corresponding to the C-Type Lectin and SP domains.

Our study of the CLSP2-mediated immune activation using RNAseq-based transcriptome analysis further supports a hypothesis that CLSP2 is a modulator of the transcription responses involved in innate immunity and suggests that CLSP2 acts upstream of extracellular pathogen-recognition factors. CLSP2 depletion affected genes encoding FREP pattern recognition receptors and TEPs indicating that CLSP2 is an immune factor working upstream of the pattern-recognition receptor system, modulating their responses.

We identified TEP22 as an important player of the anti-fungal response in *Aedes* mosquitoes. TEPs are immune effectors genes that are conserved from insects to mammals. TEP molecules contain a motif harboring an intra-chain β-cysteinyl-γ-glutamyl thioester bond, which binds to target surfaces and prompts a series of complement cascades against microbes and parasites [29]. Knockdown of AgTEP1 in the resistant strain of *An*. *gambiae* led to a massive increase in the number of *Plasmodium* oocysts [30]. AgTEP1 is essential for blocking oocyst development in the midgut of *An*. *gambiae* by forming complex with two proteins from leucine-rich repeat family, LRIM1 and APL1C [31,32,33]. Nine TEP genes have been identified in the *Ae*. *aegypti* genome [16]. Our phylogenetic analysis has shown that *Aedes* TEP20, 22, 23, and 25 form an independent clade supported by high bootstrap value (S4 Fig). Transcript levels of *TEP20*, *22*, and *23* were elevated in the fat body Rel1 and Rel2 gain-of-function transgenic mosquitoes and also in response to the *Plasmodium* infection [22]. We have shown in the present study that the *TEP22* expression is dramatically elevated in iCLSP2Bb mosquitoes. Furthermore, TEP22-depeleted mosquitoes are extremely sensitive to *B*. *bassiana* infection, while CLSP2 knockdown in these mosquitoes rescues their survival. Thus, our findings suggest that TEP22 is involved in the antifungal immune pathway and it could interact with CLSP2 in this immune response. This interaction would be reminiscent of the mannose-binding lectin (MBL) triggered complement activation in mammals [34] or TEP1/LRIM1/APL1C complex in *An*. *gambiae* [30–33]. However, the detailed mechanism of complement-like factor action in the anti-fungal immunity and TEP22 association with the immune modulating factor CLSP2 requires further mechanistic study.

Our study has demonstrated that the intracellular signal transduction components of the Toll pathway are not regulated by CLSP2 at the transcriptional level. In vertebrates, inhibitory receptor systems modulating immune responses depend on the intracellular phosphorylation pathway and not regulation at the transcription level [4,5]. Similar mechanism is also identified in the negative regulation of Toll-like receptor mediated pathways [35,36]. Interestingly, we observed that the activation of Cactus, the Rel1 inhibitor in Toll-mediated infection [37], brought by fungal infection was abolished by the RNAi depletion of CLSP2. This iCLSP2 effect on Cactus is completely opposite from those on other immune genes. Although Cactus target Rel1 is not affected by CLSP2, the downstream gene cohorts highly activated in the iCLSP2Bb mosquitoes include those encoded effector molecules such as AMPs. The unique interaction of CLSP2 with Cactus suggests that it contributes in the control of AMP gene activation. Moreover, the abolishment of activation of AMPs, brought by iCLSP2 by the double knockdown of CLSP2 and Rel1, indicates that Rel1 mediates the action of CLSP2 on these immune genes.

We also have uncovered the CLSP2 role in modulating the melanization pathway in *Ae*. *aegypti*. represents a second immune pathway that is essential in the systemic antifungal immune responses [17]. CLSP2 not only modulates the hemolymph activation of PPO, but also negatively regulates the expression of *PPO* genes. The melanization cascade is tightly regulated by serine protease inhibitors (SRPNs), which prevent spontaneous initiation of the reaction. The analysis of the mosquito genomes has shown that genes encoding immune signaling and effector molecules, and the number of melanization pathway genes have undergone major expansion [16]. For example, there are 10 PPO genes in the *Ae*. *aegypti* genome [13]. However, the precise roles of each PPO in melanization process are poorly understood. Our previous study revealed a novel level of complexity in the melanization cascade of the mosquito *Ae*. *aegypti*. Namely, we identified that there are several independent pathways leading to melanization, each requiring a different protease/SRPN regulatory module [15]. Of particular interest is a clear separation of tissue melanization, represented by melanin tumors often associated with the damage of host tissues, and immune melanization involved in the recognition and killing of pathogens, including fungi [13]. The melanization response has also been shown to significantly retard the growth and dissemination of *B*. *bassiana* in the *An*. *gambiae* mosquito [18].

Multicellular organisms have evolved complex and powerful systems of immune responses to counteract continuous attacks of various pathogens. An essential feature of the immune system in any organism is its capacity to sustain equilibrium between reactivity and quiescence [4]. A loss of such a balance leads to severe consequences, such as autoimmune and inflammatory diseases in humans. Inhibitory receptor systems balancing immune responses have been identified in vertebrates [4,5]. Our study has revealed that CLSP2 functions as a key modulator of the mosquito immune system and contributes to a better understanding of immune mechanisms in insects.

## Materials and Methods

### Experimental animals

The UGAL strain of *Ae*. *aegypti* mosquitoes was maintained in the laboratory as described previously [38]. Adults were fed continuously on water and 10% sucrose solution. To initiate egg development, mosquitoes were blood fed on chickens. All procedures for using vertebrate animals were approved by the Institute of Zoology Animal Care and Use Committee.

### Fungal culture and septic injury

*B*. *bassiana* strain ARSEF 2680 and *B*. *bassiana* strain expressed GFP were cultured on potato dextrose agar plates at 25°C and 80% humidity [39]. *B*. *bassiana* strain ARSEF 2680 was used in immune challenge and the strain 252-GFP was used in the agglutination assay. Conidia (fungal spores), used for mosquito challenge were harvested from 3- to 4-week-old cultures and diluted to 5×10^7^ conidia/ml in PBS. Septic injures were carried out by pricking the rear part of the mosquito abdomen with an acupuncture needle dipped into fungal conidia suspension [6].

For the immune response of CLSP2 to fungal infection, 3 days old adult mosquitoes were divided into two groups (30 adults / group): the control group (control) was challenged with PBS; the experiment group (Bb 24h) with *B*. *bassiana* spores. Tissue samples were collected 24 h later.

For the RNA-seq, immune genes expression and survival rate analysis, new emergence mosquitoes were divided into four groups (30 adults / group): two groups (luciferase groups) were injected with luciferase dsRNA; another two groups (CLSP2 groups) were injected with CLSP2 dsRNA. 3 days later, one of the luciferase groups were challenged with PBS (iLuc), and the other one were challenged with *B*. *bassiana* spores (iLucBb). One of the CLSP2 groups was challenged with PBS (iCLSP2), and the other one with *B*. *bassiana* spores (iCLSP2Bb). The same treatments were also used in the survival rate analysis of TEP22 and other immune genes.

### Synthesis and micro-injection of dsRNA

cDNA templates of target genes were generated by means of RT-PCR using both sense and antisense primers fused with T7-phage promoter sequences. RT-PCR was performed using the cDNA samples as templates to generate 400-bp to 1-kb gene-specific cDNA fragments. Synthesis of dsRNA was accomplished by simultaneous transcription of both strands of template DNA using T7 RNA polymerase from the T7 RiboMAX Express RNAi kit (Promega). The luciferase gene was used to generate control iLuc dsRNA. A Nanoliter 2000 injector (World Precision Instrument) was used to introduce corresponding dsRNA into the thorax of CO_2_-anesthetized mosquito females within 1 day post-eclosion. Primers used for generating dsRNA are listed in S6 Table. The transcripts of specific genes decreased to 50–70% 1 week after dsRNA injection, confirmed by real-time RT-PCR.

### Survival analysis

At 3 days after eclosion, 30 female mosquitoes were challenged with *B*. *bassiana* conidia [6]. The mosquitoes were maintained in individual containers and fed continuously on water and 10% sucrose solution. The survival curves were compared using Kaplan-Meier, and the threshold of p value was calculated with a Log-rank or Mantel Cox test, and p < 0.01 were considered to be statistically significant. Graphpad 6.0 software was used in all statistical analyses.

### Immunoblot analysis

Hemolymph from 20 decapitated mosquitoes was collected into 20 μl of 1×protease inhibitor cocktail (Roche) by centrifugation at 5,000 rpm for 5 min with Qiashredder column (QIAGEN) [15]. Aliquots of hemolymph samples were resolved on 4–15% gradients SDS-polyacrylamide gels (Bio-Rad) and electrotransferred to PVDF membranes (Invitrogen). After blocking, the membranes were incubated with the primary antibody against CLSP2 or PPO3 overnight at 4°C. We used polyclonal antibodies against *Ae*. *aegypti* Lipophorin II [15,40] and β-actin (Sigma) as the loading controls. Immune complexes were visualized by means of SuperSignal West Pico Substrate (Pierce).

### Preparation of rLectin and antibody preparation

rLectin (Lectin domain of CLSP2) was amplified by RT-PCR from cDNA with specific primers (S6 Table). The PCR product was subcloned into PSFM (a kind gift from Dr. Haobo Jiang, Oklahoma State University), a vector with a Sumo at the N-terminal, which increases the solubility of the fusion protein and can be removed by SUMO protease afterwards. The N-terminal FLAG and the C-terminal Myc are short sequences for detection of the expression of fusion protein and its cleavage products using commercially available monoclonal antibodies against these two tags, respectively. SUMO-rLectin was first purified on a Ni-NTA (nickel-nitrilotriacetic acid, Qiagen) agarose column. Then, SUMO-rLectin was cleaved using SUMO protease, as per the manufacturer’s protocol (GeneCopoeia), and re-purified on the Ni-NTA agarose column. Monoclonal antibodies were prepared against KLH-peptide from CLSP2 (Beijing Protein Innovation). Polyclonal antibodies were prepared against recombinant CLSP2 and recombinant PPO3 (Beijing Protein Innovation). Specificity tests of these antibodies are presented in S1 Fig.

### Agglutination and binding analysis of rLectin

FITC-conjugated zymosan (Molecular Probes) or GFP-conjugated *B*. *bassiana* conidia suspended in Tris-buffered saline (TBS) (25 mM Tris-HCl, 137 mM NaCl and 3 mM KCl, pH 7.0) were incubated with purified rLectin (80 μg/ml) for the agglutination assay, as described by Yu et al. [41]. After incubation for 45 min at RT, samples were examined using fluorescence confocal microscopy (Zeiss 710).

For binding assay, wells of a flat-bottom, 96-well plate (Nunc, Fisher Scientific) were coated with 2 mg (50 μl of 40 mg/ml per well) of curdlan (Sigma) as described [41]. The plate was then blocked with BSA (100 μl/well of 1 mg/ml) for 2 h at 37°C and rinsed with binding buffer (50 mM Tris-HCl, 50 mM NaCl, pH 8.0) (200 μl/well). rLectin diluted with binding buffer containing 5 mM CaCl_2_ and 0.1 mg/ml BSA was adjusted to 50 μl/well binding at RT for 4 h. The plates were rinsed as before, and bound rLectin was measured using mouse anti-C-myc antibody (1:1000), and horseradish peroxidase (HRP) conjugated antibodies against mouse. The pre-immune antiserum was used as control. Soluble TMB Substrate Solution (100 μl/well, Tiangen) was added to react for 20 min, and then stopped with 8.5 M acetic acid. Absorbance at 450 nm of the samples in each well was determined using a microplate reader (Molecular Devices).

### RNA-sequencing transcriptome experiments

To investigate the immune response to *B*. *bassiana* infection in the mosquito *Ae*. *aegypti*, we used a high-throughput sequencing (HTS) platform (HiSeq 2000) to analyze gene expression in carcasses of fungal infected mosquitoes. Four fat body libraries were built from iLucBb, iCLSP2, iCLSP2Bb, and iLuc mosquitoes. Three replicates of each sample (25 mosquitoes/sample) were pooled for analysis and 100 ng of total mRNA from each sample was used to construct libraries with an Illumina kit v2.

### Bioinformatics analysis

Raw reads generated from the sequencing were preprocessed using in-house perl scripts, including adaptor removing and low quality reads filtering. Those with average quality lower than 20 and read length shorter than 35 bp were discarded automatically. To minimize the sequencing noise from other species, we mapped the filtered reads against both the bacteria and virus databases in NCBI, and made sure the remainder was highly reliable. The genome of *Ae*. *aegypti* was downloaded from VectorBase (https://classic.vectorbase.org/genomes), as was the annotation file. The clean reads were mapped to the genome using GSNAP to estimate the expression level of all of the transcripts [42]: three mismatches were tolerated during processing, and the parameter of new transcript finding was shut down to guarantee the precise matching. We used flux-capacitor to calculate the FPKM of the transcripts, and DEGseq package in R Scripts to determine the DEGs [43]. P values less than 0.05 indicated genes were differentially expressed. All immune genes were then assigned according to immunodb [16]. Hierarchical clustering of gene expression intensity was performed using Pearson distance as the distance measure between genes and libraries [44]. Cross comparison performing within each treated sample was normalized by their reads count (FPKM), and iLuc sample was considered as background value while the ratio of fold change was calculated. Phylogenetic trees were constructed using MEGA6 by the neighbor-joining method [45].

### RNA preparation, RT-PCR, and real-time RT-PCR analysis

Total RNA samples were prepared from dissected abdominal carcasses of 10–15 individual mosquitoes. Malpighian tubules, midguts, and ovaries were removed, then abdominal carcasses with adhered fat body tissue and sessile hemocytes were rinsed in PBS, transferred into TRI reagent (Sigma), and homogenized using a motor-driven pellet pestle mixer (Kontes, Vineland, NJ). A 2-μg sample of total RNA was treated with DNase I (Invitrogen) to remove contaminating genomic DNA, and then used for cDNA synthesis (M-MLV reverse transcriptase kit, Promega). Actin was used as an internal standard to normalize the templates in a preliminary PCR experiment. After template adjustment, PCRs were performed to detect relative levels using specific primers. Primers were designed by software Primer5. Real-time RT-PCR (qPCR) reaction was performed on the MX3000P system (Stratagene, CA), and we used a SYBR green PCR Master Mix (Tiangen, Beijing) for these reactions. Thermal cycling conditions were: 94°C, 20 s; 59°C, 20 s; 68°C 20 s. Quantitative measurements were performed in triplicate and normalized to the internal control of S6 ribosomal protein mRNA for each sample. Primers and gene accession numbers are listed in S6 and S7 Tables, online. Real-time RT-PCR data were collected and exported to EXCEL for analysis. Values were represented as the mean ± SEM, and the statistically significant difference between samples was calculated using the Student-t test (Graphpad 6.0).

## Supporting Information

S1 Fig Characterization of CLSP2 and anti-CLSP2 antibodies. A) The mRNA levels of CLSP2 in response to the infection with *B*. *bassiana* conidia were measured using real-time RT-PCR. Control (control) group of mosquitoes was challenged with sterile phosphate buffered saline (PBS); Bb 24h, 24h after post-injection with *B*. *bassiana* conidia. Data are shown as mean ± SEM. **, p < 0.01. B) Purified recombinant CLSP2 and CLSP1 proteins were separated on 10% SDS-PAGE, followed by Coomassie blue staining. Approximately 80 μg of each protein purified from the nickel affinity column were loaded on the gel. C) Immunoblot evaluating the specificity of anti-CLSP2 antibodies. Left panel—Loading control utilizing the monoclonal anti-his antibody recognizing both CLSP2 and CLSP1; the middle panel—anti-CLSP2 monoclonal antibodies recognizing only CLSP2; right panel—anti-CLSP2 polyclonal antibodies predominantly recognized CLSP2 and weakly CLSP1.
(TIF)

S2 Fig Confirmation of the CLSP2 RNAi silencing efficiency and real-time RT-PCR validation of transcript levels of selected immune genes. A) The semi-quantitative RT-PCR analysis. The primers used in RT-PCR overlapped with the dsRNA corresponding region. B) Immunoblot analysis of the CLSP2 RNAi silencing using CLSP2 monoclonal antibodies. C). The real-time RT-PCR analysis of the time course reduction of the CLSP2 transcript after the CLSP2 RNAi silencing in the whole-body protein extracts of CLSP2 RNAi-depleted mosquitoes. D) Hierarchical cluster analysis of Cluster III (Fig 2A) immunity-related genes up-regulated in iCLSP2Bb mosquitoes. E) Real-time RT-PCR validation of transcript levels of selected immune genes. Data were normalized to the expression level of iLuc. iLucBb, iLuc mosquitoes infected with *B*. *bassiana*; iCLSP2Bb, CLSP2 dsRNA-treated mosquitoes infected with *B*. *bassiana*; iCLSP2, mosquitoes injected with CLSP2 dsRNA; iLuc, luciferase RNAi-treated control mosquitoes. Data were shown as mean ± SEM. * p < 0.05; ** p < 0.01.
(TIF)

S3 Fig Immune genes of IMD and JAK-STAT pathways in CLSP2 depleted mosquitoes. A) A schematic diagram of IMD and JAK/Stat pathways. Genes were not significantly affected (Ratios < 1.5 fold) are marked by green color. B) Real-time RT-PCR was performed on samples to measure the transcript level of immune genes shown in (A). Data were normalized to the expression level of iLuc. Data are shown as mean ± SEM. * p < 0.05; ** p < 0.01. iLucBb, iLuc mosquitoes infected with *B*. *bassiana*; iCLSP2Bb, CLSP2 dsRNA-treated mosquitoes infected with *B*. *bassiana*; iCLSP2, mosquitoes injected with CLSP2 dsRNA; iLuc, luciferase RNAi-treated control mosquitoes.
(TIF)

S4 Fig Phylogenetic analysis of insect TEPs. TEPs from *Ae*. *aegypti* (Aa), *An*. *gambiae* (Ag), *D*. *melanogaster* (Dm), *Tribolium castaneum* (Tc), *Apis mellifera* (Am), and *Bombyx mori* (Bm) are shown. The clade containing TEP22 is shaded. Red dots at nodes demonstrate bootstrap values above 800 out of 1000 trials. The accession numbers of proteins in the figure are listed in S7 Table.
(TIF)

S5 Fig Contribution of CLSP2 and immune genes to mosquito defense. Survival rate of mosquitoes showed that concomitant depletion of *CLSP2* and immune genes (CLIPB28, B46, B13B, B24, SPZ2, 3A, or LYSC11) did not enhance the capacity of mosquitoes to defend *B*. *bassiana* (p < 0.01) compared to single depletions of each of these genes and CLSP2 or a control. Each experiment was performed in three replicates.
(TIF)

S1 Table Repertoire of immune genes changed (fold change ≥ 1.5) in the *B*. *bassiana*-challenged mosquitoes (iLucBb). (DOCX)

S2 Table Repertoire of immune genes changed (fold change ≥ 1.5) in the CLSP2 depleted mosquitoes (iCLSP2). (DOCX)

S3 Table Repertoire of immune genes changed (fold change ≥ 1.5) in the CLSP2 dsRNA-treated mosquitoes with *B*. *bassiana* challenge (iCLSP2Bb). (DOCX)

S4 Table Up-regulated immune genes were shown in experimental conditions, iLucBb, iCLSP2 and iCLSP2Bb. (DOCX)

S5 Table RNAi for putative genes involved in the CLSP2-modulated antifungal immune response. (DOCX)

S6 Table Primers used for real-time RT-PCR, RT-PCR, dsRNA synthesis, and protein expression. (DOCX)

S7 Table The list of accession numbers/ID numbers for genes and proteins used in this work. (DOCX)
