# Supplementary figures and images for: A Critical Role for CLSP2 in the Modulation of Antifungal Immune Response in Mosquitoes
Source: PLoS Pathog. 2015 Jun 9;11(6):e1004931. doi: 10.1371/journal.ppat.1004931 (PMC4461313; doi:10.1371/journal.ppat.1004931)

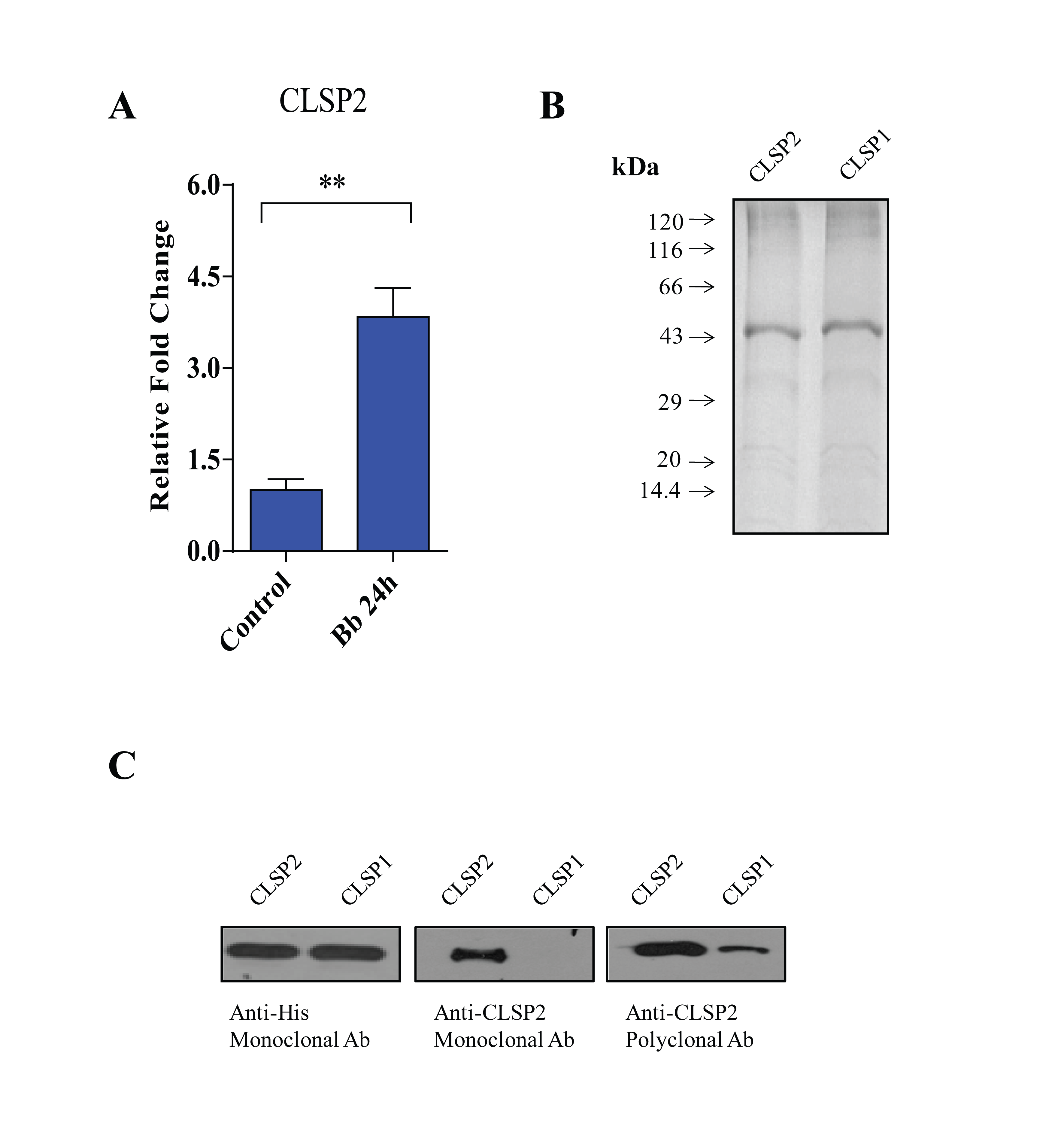

Supplement: S1 Fig — A) The mRNA levels of CLSP2 in response to the infection with B. bassiana conidia were measured using real-time RT-PCR. Control (control) group of mosquitoes was challenged with sterile phosphate buffered saline (PBS); Bb 24h, 24h after post-injection with B. bassiana conidia. Data are shown as mean ± SEM. **, p < 0.01. B) Purified recombinant CLSP2 and CLSP1 proteins were separated on 10% SDS-PAGE, followed by Coomassie blue staining. Approximately 80 μg of each protein purified from the nickel affinity column were loaded on the gel. C) Immunoblot evaluating the specificity of anti-CLSP2 antibodies. Left panel—Loading control utilizing the monoclonal anti-his antibody recognizing both CLSP2 and CLSP1; the middle panel—anti-CLSP2 monoclonal antibodies recognizing only CLSP2; right panel—anti-CLSP2 polyclonal antibodies predominantly recognized CLSP2 and weakly CLSP1. (TIF) [file ppat.1004931.s001.tif]

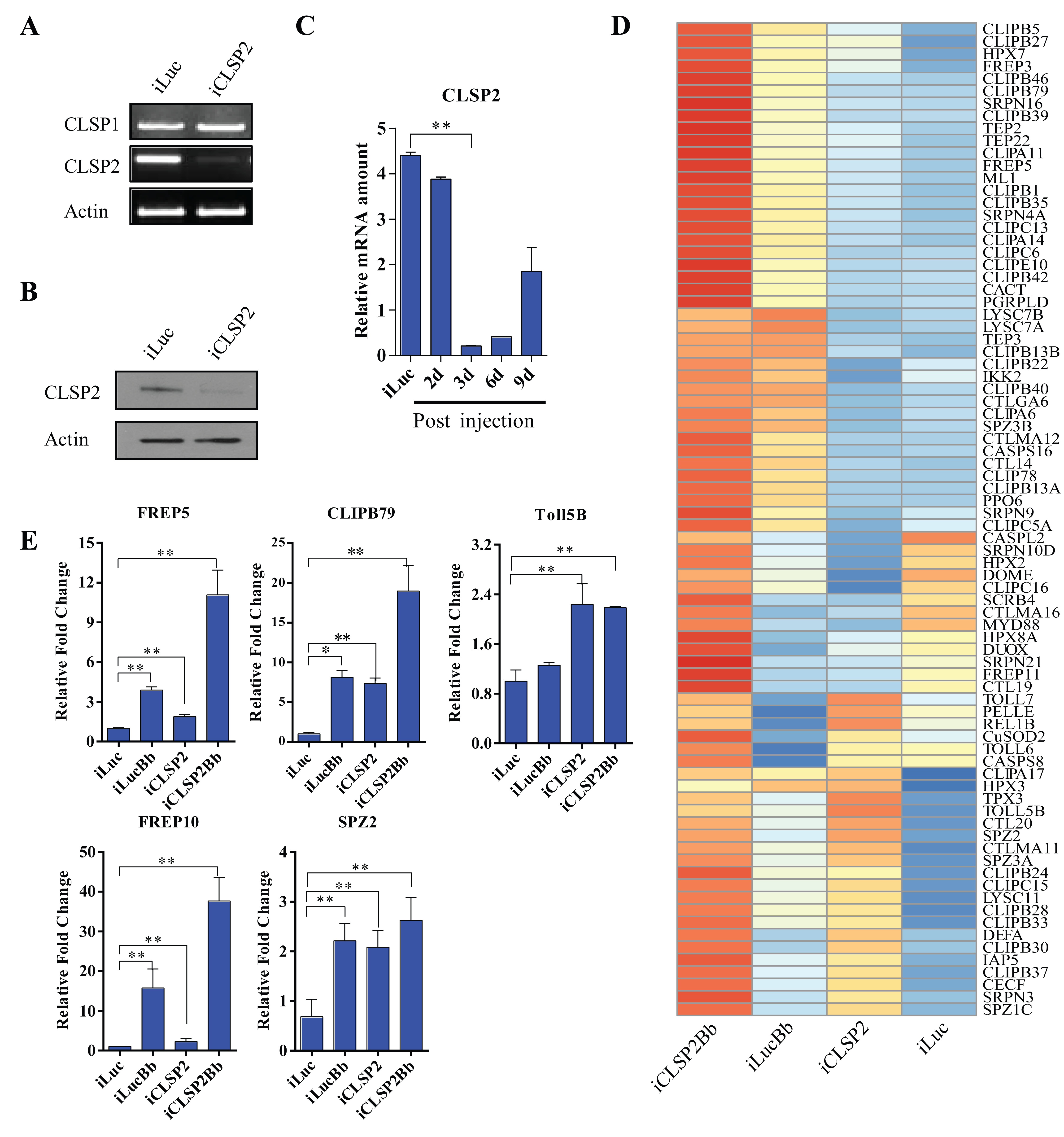

Supplement: S2 Fig — A) The semi-quantitative RT-PCR analysis. The primers used in RT-PCR overlapped with the dsRNA corresponding region. B) Immunoblot analysis of the CLSP2 RNAi silencing using CLSP2 monoclonal antibodies. C). The real-time RT-PCR analysis of the time course reduction of the CLSP2 transcript after the CLSP2 RNAi silencing in the whole-body protein extracts of CLSP2 RNAi-depleted mosquitoes. D) Hierarchical cluster analysis of Cluster III (Fig 2A) immunity-related genes up-regulated in iCLSP2Bb mosquitoes. E) Real-time RT-PCR validation of transcript levels of selected immune genes. Data were normalized to the expression level of iLuc. iLucBb, iLuc mosquitoes infected with B. bassiana; iCLSP2Bb, CLSP2 dsRNA-treated mosquitoes infected with B. bassiana; iCLSP2, mosquitoes injected with CLSP2 dsRNA; iLuc, luciferase RNAi-treated control mosquitoes. Data were shown as mean ± SEM. * p < 0.05; ** p < 0.01. (TIF) [file ppat.1004931.s002.tif]

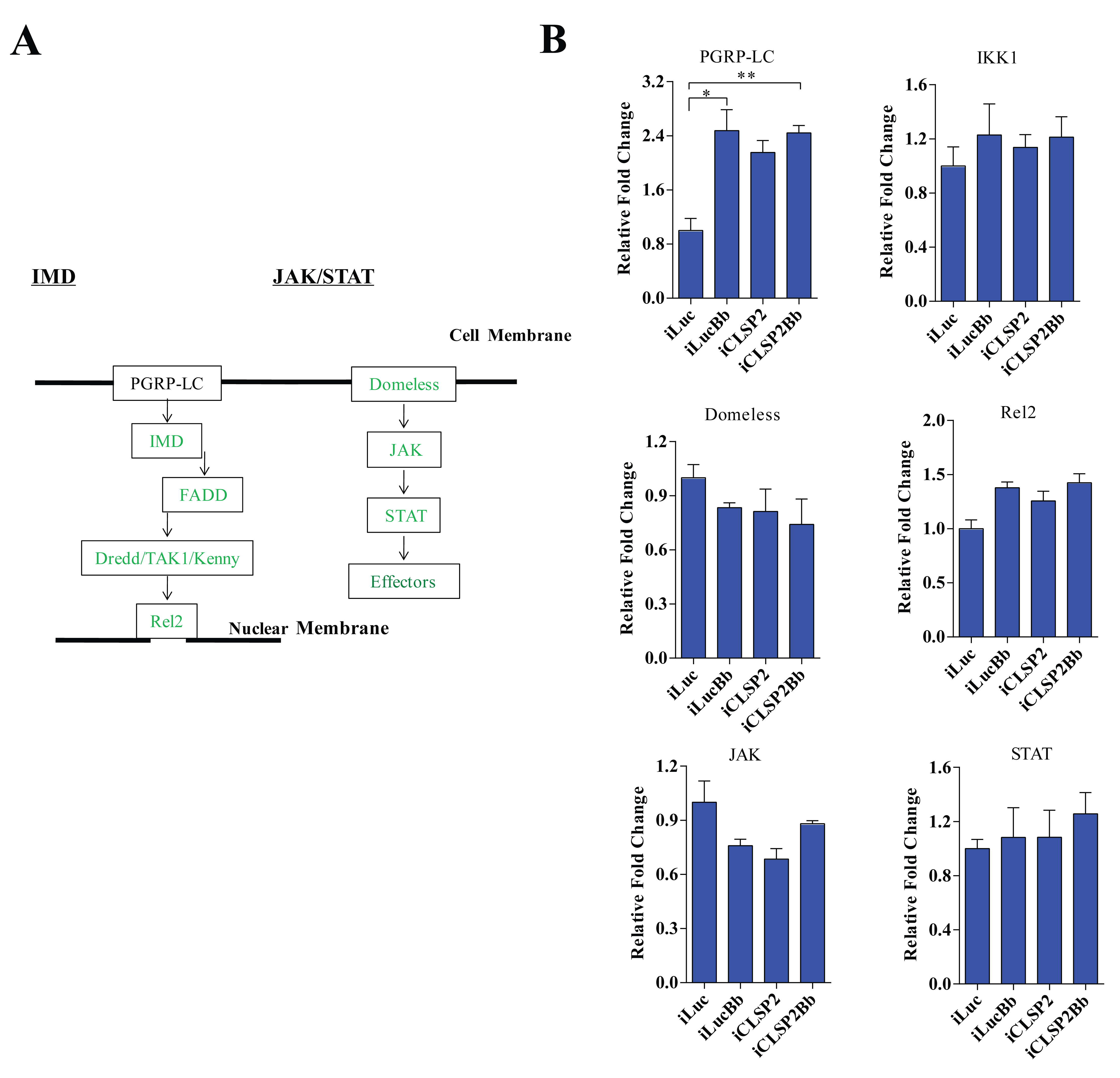

Supplement: S3 Fig — A) A schematic diagram of IMD and JAK/Stat pathways. Genes were not significantly affected (Ratios < 1.5 fold) are marked by green color. B) Real-time RT-PCR was performed on samples to measure the transcript level of immune genes shown in (A). Data were normalized to the expression level of iLuc. Data are shown as mean ± SEM. * p < 0.05; ** p < 0.01. iLucBb, iLuc mosquitoes infected with B. bassiana; iCLSP2Bb, CLSP2 dsRNA-treated mosquitoes infected with B. bassiana; iCLSP2, mosquitoes injected with CLSP2 dsRNA; iLuc, luciferase RNAi-treated control mosquitoes. (TIF) [file ppat.1004931.s003.tif]

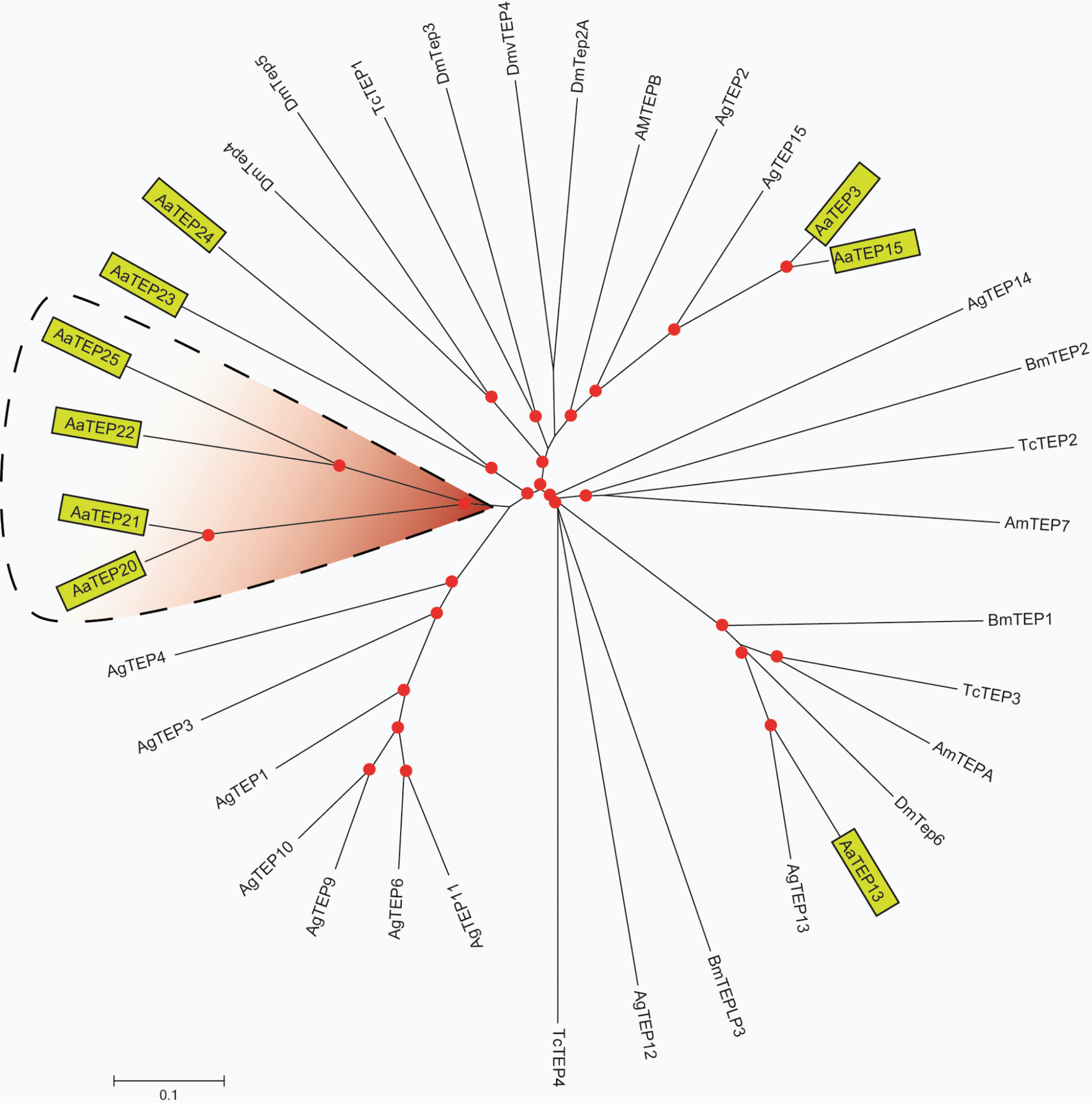

Supplement: S4 Fig — TEPs from Ae. aegypti (Aa), An. gambiae (Ag), D. melanogaster (Dm), Tribolium castaneum (Tc), Apis mellifera (Am), and Bombyx mori (Bm) are shown. The clade containing TEP22 is shaded. Red dots at nodes demonstrate bootstrap values above 800 out of 1000 trials. The accession numbers of proteins in the figure are listed in S7 Table. (TIF) [file ppat.1004931.s004.tif]

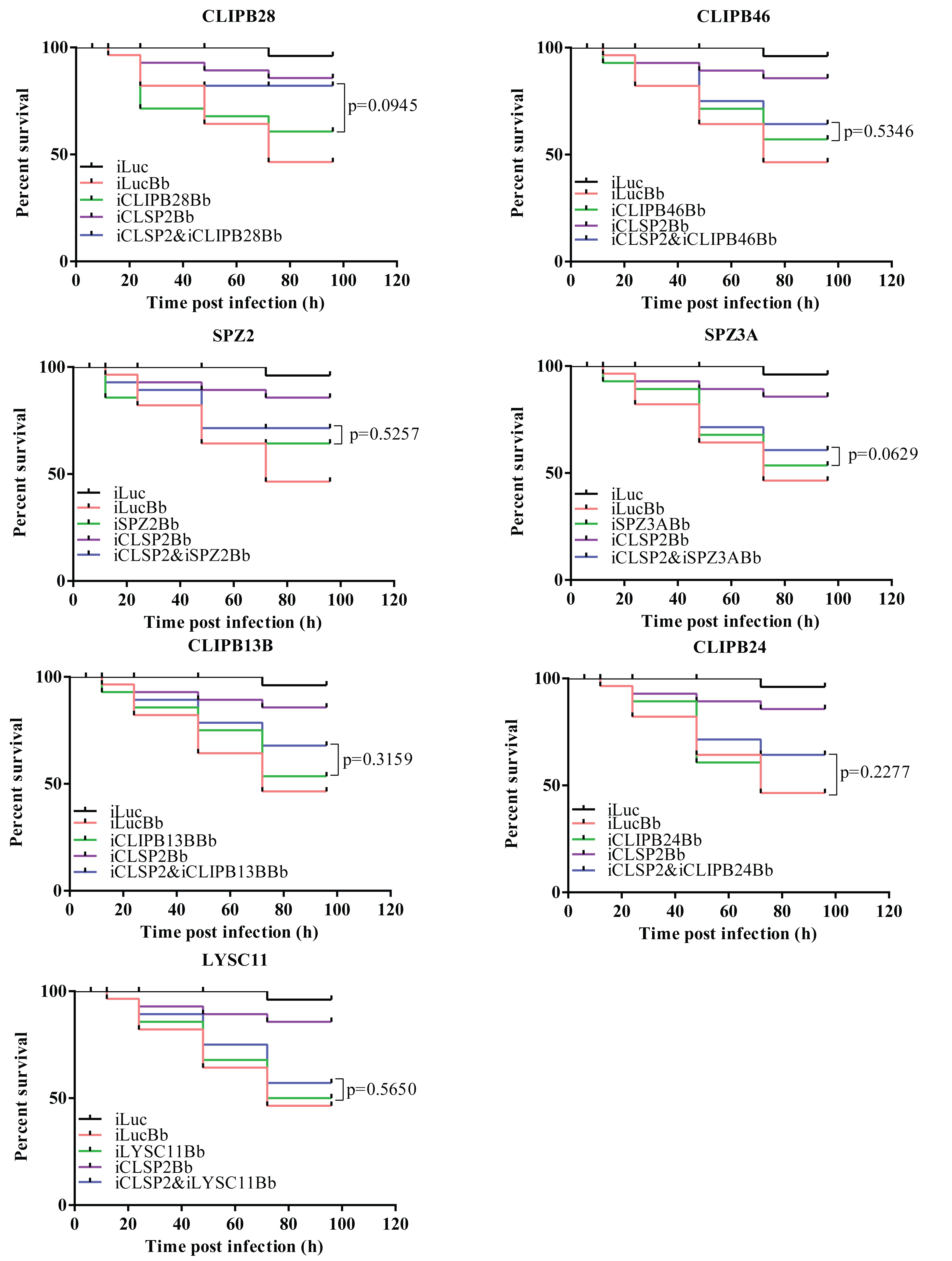

Supplement: S5 Fig — Survival rate of mosquitoes showed that concomitant depletion of CLSP2 and immune genes (CLIPB28, B46, B13B, B24, SPZ2, 3A, or LYSC11) did not enhance the capacity of mosquitoes to defend B. bassiana (p < 0.01) compared to single depletions of each of these genes and CLSP2 or a control. Each experiment was performed in three replicates. (TIF) [file ppat.1004931.s005.tif]
