# Supplementary material for: A Critical Role for CLSP2 in the Modulation of Antifungal Immune Response in Mosquitoes
Source: PLoS Pathog. 2015 Jun 9;11(6):e1004931. doi: 10.1371/journal.ppat.1004931 (PMC4461313; doi:10.1371/journal.ppat.1004931)
Supplement: S1 Table — (DOCX) [file ppat.1004931.s006.docx]

Table S1. Repertoire of immune genes changed (fold change ≥ 1.5) in the *B. bassiana*-challenged mosquitoes (iLucBb).

| Gene ID | Name | Fold change |
| --- | --- | --- |
| AAEL011455 | CTLMA12 | 20.7 |
| AAEL014139 | CLIPB79 | 16.3 |
| AAEL008646 | FREP10 | 13.1 |
| AAEL003389 | ATT | 13.1 |
| AAEL014138 | SRPN16 | 9.8 |
| AAEL002585 | CLIPA11 | 8.2 |
| AAEL005792 | CLIPE8 | 7.1 |
| AAEL012712 | CLIPC13 | 6.9 |
| AAEL003632 | CLIPB39 | 6.7 |
| AAEL003294 | FREP3 | 6.6 |
| AAEL002720 | SRPN20 | 6.4 |
| AAEL003253 | CLIPB13B | 6.3 |
| AAEL009384 | FREP5 | 6.1 |
| AAEL007993 | CLIPB27 | 5.6 |
| AAEL012353 | CTL15 | 5.2 |
| AAEL003697 | SRPN17 | 5.2 |
| AAEL005093 | CLIPB46 | 5 |
| AAEL011446 | CTL17 | 5 |
| AAEL005482 | CTL18 | 4.7 |
| AAEL003614 | CLIPB40 | 4.7 |
| AAEL008607 | TEP3 | 4.3 |
| AAEL010100 | LYSC7A | 3.7 |
| AAEL015515 | CECG | 3.6 |
| AAEL011610 | CTLGA7 | 3.6 |
| AAEL007992 | CLIPB78 | 3.5 |
| AAEL011453 | CTL14 | 3.5 |
| AAEL000625 | CECF | 3.2 |
| AAEL000563 | CTLMA15 | 3.1 |
| AAEL006674 | CLIPB29 | 3 |
| AAEL002288 | CLIPA4 | 2.9 |
| AAEL014140 | CLIPB24 | 2.9 |
| AAEL000037 | CLIPB35 | 2.8 |
| AAEL007006 | CLIPA17 | 2.8 |
| AAEL002524 | CTL24 | 2.8 |
| AAEL014078 | SRPN2 | 2.8 |
| AAEL000087 | TEP22 | 2.7 |
| AAEL010773 | CLIPE10 | 2.7 |
| AAEL000508 | FREP15 | 2.7 |
| AAEL013417 | FREP24 | 2.6 |
| AAEL009436 | CuSOD | 2.6 |
| AAEL000057 | TOLL5B | 2.5 |
| AAEL014755 | TEP2 | 2.5 |
| AAEL000074 | CLIPB1 | 2.5 |
| AAEL013245 | CLIPB28 | 2.5 |
| AAEL014390 | CTL | 2.4 |
| AAEL011070 | CTLGA3 | 2.4 |
| AAEL000028 | CLIPB34 | 2.4 |
| AAEL000059 | CLIPB19 | 2.4 |
| AAEL002731 | SRPN14 | 2.3 |
| AAEL003243 | CLIPB13A | 2.3 |
| AAEL002595 | CLIPA14 | 2.3 |
| AAEL003642 | CLIPB10 | 2.3 |
| AAEL002301 | CLIPA5 | 2.1 |
| AAEL014137 | CLIPB25 | 2.2 |
| AAEL014148 | CASPL1 | 2.2 |
| AAEL015404 | LYSC7B | 2.1 |
| AAEL000611 | CECE | 2.1 |
| AAEL003723 | LYSC11 | 2.1 |
| AAEL005988 | LYSC6 | 1.9 |
| AAEL003625 | CLIPB8 | 1.9 |
| AAEL005416 | HPX3 | 1.9 |
| AAEL002629 | CLIPA6 | 1.9 |
| AAEL012135 | GALE2 | 1.9 |
| AAEL000495 | GPXH3 | 1.9 |
| AAEL004540 | CLIPC6 | 1.9 |
| AAEL006168 | CLIPB42 | 1.8 |
| AAEL000556 | CTL25 | 1.8 |
| AAEL004120 | ML1 | 1.8 |
| AAEL005064 | CLIPB5 | 1.8 |
| AAEL003841 | DEFA | 1.5 |
| AAEL000749 | FREP22 | 1.7 |
| AAEL005648 | CLIPB16 | 1.7 |
| AAEL008596 | SPZ3A | 1.7 |
| AAEL002354 | HPX5 | 1.7 |
| AAEL003933 | DBLOX | 1.7 |
| AAEL000238 | CLIPD9 | 1.7 |
| AAEL001435 | SPZ2 | 1.7 |
| AAEL007420 | SRPN25 | 1.7 |
| AAEL005956 | CASPS16 | 1.7 |
| AAEL013936 | SRPN4A | 1.7 |
| AAEL003610 | CLIPB9 | 1.7 |
| AAEL000726 | FREP20 | 1.7 |
| AAEL014640 | PGRP-LC | 1.6 |
| AAEL004524 | CLIPC5B | 1.6 |
| AAEL003245 | IKK2 | 1.6 |
| AAEL014354 | CLIPB43 | 1.5 |
| AAEL003182 | SRPN26 | 1.5 |
| AAEL003849 | DEFE | 1.5 |
| AAEL008364 | SRPN9 | 1.5 |
| AAEL010769 | SRPN6 | 1.5 |
| AAEL002704 | SRPN23 | 1.5 |
| AAEL000709 | CACT | 1.5 |
| AAEL012380 | PGRP-LA | 1.5 |
| AAEL000671 | TOLL6 | 0.6 |
| AAEL003844 | GALE5 | 0.6 |
| AAEL003832 | DEFC | 0.6 |
| AAEL003631 | CLIPB41 | 0.6 |
| AAEL002126 | CLIPA15 | 0.6 |
| AAEL011612 | CTLMA6 | 0.5 |
| AAEL002741 | SCRB6 | 0.5 |
| AAEL000633 | TOLL8 | 0.5 |
| AAEL003444 | CASPS19 | 0.5 |
| AAEL010131 | FREP1 | 0.5 |
| AAEL006161 | CLIPB31 | 0.5 |
| AAEL014348 | CASPS8 | 0.5 |
| AAEL000283 | CTLMA16 | 0.4 |
| AAEL009423 | SCRBQ2 | 0.4 |
| AAEL013566 | CTLGA2 | 0.4 |
| AAEL009474 | PGRPS1 | 0.4 |
| AAEL008404 | CLIPA16 | 0.3 |
| AAEL006014 | HPX1 | 0.3 |
| AAEL005641 | CTLGA5 | 0.2 |
| AAEL012481 | HPX6 | 0.1 |

Ratio of fold change was calculated from FPKM of iLucBb/FPKM of iLuc.
