# Supplementary material for: A Critical Role for CLSP2 in the Modulation of Antifungal Immune Response in Mosquitoes
Source: PLoS Pathog. 2015 Jun 9;11(6):e1004931. doi: 10.1371/journal.ppat.1004931 (PMC4461313; doi:10.1371/journal.ppat.1004931)
Supplement: S2 Table — (DOCX) [file ppat.1004931.s007.docx]

Table S2. Repertoire of immune genes changed (fold change ≥ 1.5) in the CLSP2 depleted mosquitoes (iCLSP2).

| Gene ID | Name | Fold change |
| --- | --- | --- |
| AAEL015515 | CECG | 15.9 |
| AAEL003841 | DEFA | 8.4 |
| AAEL003610 | CLIPB9 | 7.1 |
| AAEL000625 | CECF | 7.1 |
| AAEL003857 | DEFD | 6.1 |
| AAEL003832 | DEFC | 5.4 |
| AAEL000057 | TOLL5B | 4.8 |
| AAEL007993 | CLIPB27 | 4.6 |
| AAEL008646 | FREP10 | 4.4 |
| AAEL003294 | FREP3 | 4.3 |
| AAEL012353 | CTL15 | 3.7 |
| AAEL005648 | CLIPB16 | 3.6 |
| AAEL014140 | CLIPB24 | 3.5 |
| AAEL002585 | CLIPA11 | 3.4 |
| AAEL001435 | SPZ2 | 3.3 |
| AAEL000621 | CECN | 3.3 |
| AAEL007006 | CLIPA17 | 3.2 |
| AAEL013245 | CLIPB28 | 3.2 |
| AAEL000563 | CTLMA15 | 2.9 |
| AAEL000611 | CECE | 2.9 |
| AAEL003723 | LYSC11 | 2.8 |
| AAEL005988 | LYSC6 | 2.5 |
| AAEL008596 | SPZ3A | 2.5 |
| AAEL003697 | SRPN17 | 2.5 |
| AAEL013499 | PPO2 | 2.4 |
| AAEL009384 | FREP5 | 2.4 |
| AAEL013417 | FREP24 | 2.4 |
| AAEL014138 | SRPN16 | 2.4 |
| AAEL014390 | CTL | 2.4 |
| AAEL005792 | CLIPE8 | 2.3 |
| AAEL000238 | CLIPD9 | 2.3 |
| AAEL014139 | CLIPB79 | 2.2 |
| AAEL009436 | CuSOD | 2.1 |
| AAEL009338 | CTL10 | 2 |
| AAEL003253 | CLIPB13B | 1.9 |
| AAEL011400 | FREP36 | 1.9 |
| AAEL014148 | CASPL1 | 1.9 |
| AAEL005416 | HPX3 | 1.9 |
| AAEL002731 | SRPN14 | 1.9 |
| AAEL000543 | CTLMA11 | 1.9 |
| AAEL000087 | TEP22 | 1.8 |
| AAEL012712 | CLIPC13 | 1.8 |
| AAEL013498 | PPO1 | 1.8 |
| AAEL005431 | CLIPB37 | 1.8 |
| AAEL011446 | CTL17 | 1.8 |
| AAEL005482 | CTL18 | 1.7 |
| AAEL002720 | SRPN20 | 1.7 |
| AAEL005093 | CLIPB46 | 1.7 |
| AAEL006674 | CLIPB29 | 1.7 |
| AAEL014078 | SRPN2 | 1.7 |
| AAEL005416 | HPX3 | 1.6 |
| AAEL003642 | CLIPB10 | 1.7 |
| AAEL014079 | SRPN1 | 1.7 |
| AAEL000749 | FREP22 | 1.7 |
| AAEL003625 | CLIPB8 | 1.6 |
| AAEL008397 | GPXH2 | 1.6 |
| AAEL014432 | FREP25 | 1.6 |
| AAEL011763 | PPO3 | 1.7 |
| AAEL007420 | SRPN25 | 1.6 |
| AAEL014755 | TEP2 | 1.6 |
| AAEL000556 | CTL25 | 1.6 |
| AAEL012135 | GALE2 | 1.6 |
| AAEL004524 | CLIPC5B | 1.5 |
| AAEL001084 | CLIPB21 | 1.5 |
| AAEL012711 | CLIPC12 | 1.5 |
| AAEL010270 | CLIPC15 | 1.7 |
| AAEL014640 | PGRPLC | 1.5 |
| AAEL000227 | SCRB8 | 1.5 |
| AAEL014354 | CLIPB43 | 1.5 |
| AAEL011610 | CTLGA7 | 1.5 |
| AAEL000760 | CLIPB30 | 1.5 |
| AAEL002288 | CLIPA4 | 1.5 |
| AAEL001233 | CLIPE9 | 0.6 |
| AAEL006014 | HPX1 | 0.6 |
| AAEL004948 | CLIPC14 | 0.6 |
| AAEL006161 | CLIPB31 | 0.6 |
| AAEL007613 | TOLL1A | 0.6 |
| AAEL005194 | FREP26 | 0.6 |
| AAEL015439 | CLIPD7 | 0.5 |
| AAEL012069 | GPXH1 | 0.5 |
| AAEL009423 | SCRBQ2 | 0.5 |
| AAEL009760 | ML21 | 0.5 |
| AAEL007897 | SPZ4 | 0.5 |
| AAEL000283 | CTLMA16 | 0.5 |
| AAEL009420 | SCRBQ1 | 0.5 |
| AAEL009845 | GALE13 | 0.5 |
| AAEL005963 | CASPS15 | 0.5 |
| AAEL010171 | PGRPLB | 0.5 |
| AAEL006704 | FREP18 | 0.4 |
| AAEL013501 | PPO4 | 0.4 |
| AAEL006702 | FREP33 | 0.4 |
| AAEL003614 | CLIPB40 | 0.4 |
| AAEL011777 | SRPN8 | 0.4 |
| AAEL010083 | IMD | 0.4 |
| AAEL008404 | CLIPA16 | 0.4 |
| AAEL012064 | ML2 | 0.4 |
| AAEL006371 | CLIPB47-76 | 0.3 |
| AAEL003653 | SRPN12 | 0.3 |
| AAEL004833 | DPT | 0.3 |
| AAEL006699 | FREP34 | 0.3 |
| AAEL011621 | CTLMA13 | 0.3 |
| AAEL005641 | CTLGA5 | 0.3 |
| AAEL009850 | GALE14 | 0.3 |
| AAEL003444 | CASPS19 | 0.3 |
| AAEL011633 | FREP16 | 0.3 |

Ratio of fold change was calculated from FPKM of iCLSP2/FPKM of iLuc.
