# Supplementary material for: A Critical Role for CLSP2 in the Modulation of Antifungal Immune Response in Mosquitoes
Source: PLoS Pathog. 2015 Jun 9;11(6):e1004931. doi: 10.1371/journal.ppat.1004931 (PMC4461313; doi:10.1371/journal.ppat.1004931)
Supplement: S3 Table — (DOCX) [file ppat.1004931.s008.docx]

Table S3. Repertoire of immune genes changed (fold change ≥ 1.5) in the CLSP2 dsRNA-treated mosquitoes with B. bassiana challenge (iCLSP2Bb).

| Gene ID | Name | Fold change |
| --- | --- | --- |
| AAEL014139 | CLIPB79 | 36.6 |
| AAEL011455 | CTLMA12 | 34.4 |
| AAEL014138 | SRPN16 | 26.3 |
| AAEL002585 | CLIPA11 | 19.6 |
| AAEL003632 | CLIPB39 | 17.9 |
| AAEL008646 | FREP10 | 17.8 |
| AAEL012712 | CLIPC13 | 13 |
| AAEL009384 | FREP5 | 12.5 |
| AAEL003294 | FREP3 | 12.3 |
| AAEL005792 | CLIPE8 | 10.2 |
| AAEL005093 | CLIPB46 | 10.2 |
| AAEL000059 | CLIPB19 | 9.8 |
| AAEL007993 | CLIPB27 | 9.6 |
| AAEL000625 | CECF | 9.5 |
| AAEL013499 | PPO2 | 8.6 |
| AAEL000563 | CTLMA15 | 7.9 |
| AAEL002720 | SRPN20 | 7.9 |
| AAEL011446 | CTL17 | 6.4 |
| AAEL003253 | CLIPB13B | 6.3 |
| AAEL012353 | CTL15 | 5.9 |
| AAEL000087 | TEP22 | 5.9 |
| AAEL014755 | TEP2 | 5.8 |
| AAEL010773 | CLIPE10 | 5.6 |
| AAEL014140 | CLIPB24 | 5.4 |
| AAEL003697 | SRPN17 | 5.4 |
| AAEL003614 | CLIPB40 | 4.9 |
| AAEL007992 | CLIPB78 | 4.8 |
| AAEL000507 | Peroxidase | 4.7 |
| AAEL000037 | CLIPB35 | 4.5 |
| AAEL011453 | CTL14 | 4.3 |
| AAEL008607 | TEP3 | 4.3 |
| AAEL000074 | CLIPB1 | 4 |
| AAEL000057 | TOLL5B | 4 |
| AAEL013245 | CLIPB28 | 4 |
| AAEL010769 | SRPN6 | 3.5 |
| AAEL006674 | CLIPB29 | 3.5 |
| AAEL003610 | CLIPB9 | 3.4 |
| AAEL003723 | LYSC11 | 3.4 |
| AAEL002595 | CLIPA14 | 3.3 |
| AAEL010100 | LYSC7A | 3.3 |
| AAEL000508 | FREP15 | 3.3 |
| AAEL006168 | CLIPB42 | 3.3 |
| AAEL001435 | SPZ2 | 3.3 |
| AAEL007006 | CLIPA17 | 3.2 |
| AAEL011408 | CTL21 | 3.1 |
| AAEL003243 | CLIPB13A | 3 |
| AAEL014141 | SRPN5 | 2.9 |
| AAEL004120 | ML1 | 2.9 |
| AAEL000028 | CLIPB34 | 2.8 |
| AAEL008596 | SPZ3A | 2.8 |
| AAEL005416 | HPX3 | 1.6 |
| AAEL004540 | CLIPC6 | 2.8 |
| AAEL011621 | CTLMA13 | 2.7 |
| AAEL003642 | CLIPB10 | 2.7 |
| AAEL005648 | CLIPB16 | 2.4 |
| AAEL002288 | CLIPA4 | 2.3 |
| AAEL013936 | SRPN4A | 2.3 |
| AAEL008364 | SRPN9 | 2.3 |
| AAEL002301 | CLIPA5 | 2.3 |
| AAEL014078 | SRPN2 | 2.3 |
| AAEL005064 | CLIPB5 | 2.3 |
| AAEL003389 | ATT | 2.2 |
| AAEL000709 | CACT | 2.2 |
| AAEL011610 | CTLGA7 | 2.2 |
| AAEL002629 | CLIPA6 | 2.2 |
| AAEL005431 | CLIPB37 | 2.1 |
| AAEL011404 | CTL19 | 2.1 |
| AAEL005956 | CASPS16 | 2.1 |
| AAEL000611 | CECE | 2.1 |
| AAEL009850 | GALE14 | 2 |
| AAEL003841 | DEFA | 10.5 |
| AAEL005482 | CTL18 | 2 |
| AAEL000543 | CTLMA11 | 1.9 |
| AAEL007619 | TOLL5A | 1.9 |
| AAEL015404 | LYSC7B | 1.9 |
| AAEL014658 | CASPS20 | 1.8 |
| AAEL011070 | CTLGA3 | 1.8 |
| AAEL011608 | PGRP-LD | 1.8 |
| AAEL014148 | CASPL1 | 1.8 |
| AAEL000621 | CECN | 1.8 |
| AAEL011777 | SRPN8 | 1.8 |
| AAEL014354 | CLIPB43 | 1.7 |
| AAEL009436 | CuSOD | 1.7 |
| AAEL010270 | CLIPC15 | 1.7 |
| AAEL004518 | CLIPC5A | 1.7 |
| AAEL002730 | SRPN21 | 1.7 |
| AAEL000760 | CLIPB30 | 1.6 |
| AAEL014356 | CTLSE2 | 1.6 |
| AAEL002601 | CLIPA1 | 1.6 |
| AAEL012380 | PGRP-LA | 1.6 |
| AAEL006854 | ML13 | 1.5 |
| AAEL003245 | IKK1 | 1.5 |
| AAEL014137 | CLIPB25 | 1.5 |
| AAEL011402 | CTL26 | 1.5 |
| AAEL008668 | CLIPB22 | 1.5 |
| AAEL008370 | SCRBQ4 | 1.5 |
| AAEL014349 | CLIPB15 | 1.5 |
| AAEL013433 | SPZ1C | 1.5 |
| AAEL004223 | CECB | 1.5 |
| AAEL004823 | MnSOD1 | 1.5 |
| AAEL004979 | CLIPD2 | 1.7 |
| AAEL005108 | MnSOD2 | 3.5 |
| AAEL005718 | CLIPA3 | 2.1 |
| AAEL003889 | GNBP1 | 1.7 |
| AAEL009551 | TOLL11 | 1.5 |
| AAEL009670 | LYSC9 | 1.5 |
| AAEL009845 | GALE13 | 1.5 |
| AAEL011498 | CuSOD3 | 1.6 |
| AAEL006161 | CLIPB31 | 0.6 |
| AAEL005988 | LYSC6 | 0.6 |
| AAEL003541 | GALE1 | 0.6 |
| AAEL002731 | SRPN14 | 0.6 |
| AAEL002309 | TPX4 | 0.6 |
| AAEL004223 | CECB | 0.6 |
| AAEL013566 | CTLGA2 | 0.6 |
| AAEL001929 | SPZ5 | 0.6 |
| AAEL013441 | TOLL9A | 0.5 |
| AAEL002126 | CLIPA15 | 0.5 |
| AAEL000256 | SCRB9 | 0.5 |
| AAEL013417 | FREP24 | 0.5 |
| AAEL005641 | CTLGA5 | 0.5 |
| AAEL011222 | SCRB5 | 0.5 |
| AAEL004156 | FREP9 | 0.5 |
| AAEL004833 | DPT | 0.5 |
| AAEL001084 | CLIPB21 | 0.5 |
| AAEL008404 | CLIPA16 | 0.5 |
| AAEL007942 | FREP14 | 0.5 |
| AAEL000495 | GPXH3 | 0.5 |
| AAEL000749 | FREP22 | 0.5 |
| AAEL009474 | PGRPS1 | 0.4 |
| AAEL014091 | CuSOD1 | 0.4 |
| AAEL010131 | FREP1 | 0.4 |
| AAEL011009 | FREP13 | 0.4 |
| AAEL002704 | SRPN23 | 0.4 |
| AAEL009432 | SCRBQ3 | 0.4 |
| AAEL000533 | CTL16 | 0.4 |
| AAEL014432 | FREP25 | 0.4 |
| AAEL000556 | CTL25 | 0.4 |
| AAEL003933 | DBLOX | 0.4 |
| AAEL002741 | SCRB6 | 0.4 |
| AAEL001233 | CLIPE9 | 0.4 |
| AAEL012481 | HPX6 | 0.3 |
| AAEL007420 | SRPN25 | 0.3 |
| AAEL000726 | FREP20 | 0.3 |
| AAEL009842 | GALE12 | 0.3 |
| AAEL003444 | CASPS19 | 0.3 |
| AAEL003182 | SRPN26 | 0.3 |
| AAEL012064 | ML2 | 0.3 |
| AAEL015308 | SCRAL1 | 0.2 |
| AAEL011400 | FREP36 | 0.2 |
| AAEL006702 | FREP33 | 0.2 |
| AAEL008929 | CTLSE1 | 0.2 |
| AAEL015136 | ML6 | 0.2 |

Ratio of fold change was calculated from FPKM of iCLSP2Bb/FPKM of iLuc.
