# Supplementary material for: A Critical Role for CLSP2 in the Modulation of Antifungal Immune Response in Mosquitoes
Source: PLoS Pathog. 2015 Jun 9;11(6):e1004931. doi: 10.1371/journal.ppat.1004931 (PMC4461313; doi:10.1371/journal.ppat.1004931)
Supplement: S4 Table — (DOCX) [file ppat.1004931.s009.docx]

Table Fold changes of up-regulated immune genes in iLucBb, iCLSP2 and iCLSP2Bb.

| Gene ID | Bb | iCLSP2 | iCLSP2Bb | Name |
| --- | --- | --- | --- | --- |
| AAEL000057 | 2.5 | 4.8 | 4.0 | TOLL5B |
| AAEL000087 | 2.7 | 1.8 | 5.9 | TEP22 |
| AAEL000563 | 3.1 | 2.9 | 7.9 | CTLMA15 |
| AAEL000611 | 2.1 | 2.9 | 2.1 | CECE |
| AAEL000625 | 3.2 | 7.1 | 9.5 | CECF |
| AAEL001435 | 1.7 | 3.3 | 3.3 | SPZ2 |
| AAEL002288 | 2.9 | 1.5 | 2.3 | CLIPA4 |
| AAEL005416 | 1.9 | 1.9 | 1.6 | HPX3 |
| AAEL002585 | 8.2 | 3.4 | 19.6 | CLIPA11 |
| AAEL002720 | 6.4 | 1.7 | 7.9 | SRPN20 |
| AAEL003253 | 6.3 | 1.9 | 6.3 | CLIPB13B |
| AAEL003294 | 6.6 | 4.3 | 12.3 | FREP3 |
| AAEL003610 | 1.7 | 7.1 | 3.4 | CLIPB9 |
| AAEL003642 | 2.3 | 1.7 | 2.7 | CLIPB10 |
| AAEL003697 | 5.2 | 2.5 | 5.4 | SRPN17 |
| AAEL003723 | 2.0 | 2.8 | 3.4 | LYSC11 |
| AAEL005093 | 5.0 | 1.7 | 10.2 | CLIPB46 |
| AAEL005482 | 4.7 | 1.7 | 2.0 | CTL18 |
| AAEL005648 | 1.7 | 3.6 | 2.4 | CLIPB16 |
| AAEL005792 | 7.1 | 2.3 | 10.2 | CLIPE8 |
| AAEL006674 | 3.0 | 1.7 | 3.5 | CLIPB29 |
| AAEL007006 | 2.8 | 3.2 | 3.2 | CLIPA17 |
| AAEL007993 | 5.6 | 4.6 | 9.6 | CLIPB27 |
| AAEL008596 | 1.7 | 2.5 | 2.8 | SPZ3A |
| AAEL008646 | 13.1 | 4.4 | 17.8 | FREP10 |
| AAEL009384 | 6.1 | 2.4 | 12.5 | FREP5 |
| AAEL009436 | 2.6 | 2.0 | 1.7 | CuSOD |
| AAEL011446 | 5.0 | 1.8 | 6.4 | CTL17 |
| AAEL011610 | 3.6 | 1.5 | 2.2 | CTLGA7 |
| AAEL012353 | 5.2 | 3.7 | 5.9 | CTL15 |
| AAEL012712 | 6.9 | 1.8 | 13.0 | CLIPC13 |
| AAEL013245 | 2.5 | 3.2 | 4.0 | CLIPB28 |
| AAEL014078 | 2.8 | 1.7 | 2.3 | SRPN2 |
| AAEL014138 | 9.8 | 2.4 | 26.3 | SRPN16 |
| AAEL014139 | 16.3 | 2.2 | 36.6 | CLIPB79 |
| AAEL014140 | 2.9 | 3.5 | 5.4 | CLIPB24 |
| AAEL014148 | 2.1 | 1.9 | 1.8 | CASPL1 |
| AAEL014354 | 1.5 | 1.5 | 1.7 | CLIPB43 |
| AAEL003841 | 1.5 | 8.4 | 10.5 | DEFA |
| AAEL014755 | 2.5 | 1.6 | 5.8 | TEP2 |

Ratio of fold change was calculated from FPKM of treated sample/FPKM of control sample (iLuc).
