# Supplementary material for: A Critical Role for CLSP2 in the Modulation of Antifungal Immune Response in Mosquitoes
Source: PLoS Pathog. 2015 Jun 9;11(6):e1004931. doi: 10.1371/journal.ppat.1004931 (PMC4461313; doi:10.1371/journal.ppat.1004931)
Supplement: S5 Table — (DOCX) [file ppat.1004931.s010.docx]

Table S5 RNA interference for the putative genes involved in the CLSP2 modulated antifungal immune response.

| Gene id | Name |
| --- | --- |
| AAEL014140 | CLIPB24 |
| AAEL003253 | CLIPB13B |
| AAEL005093 | CLIPB46 |
| AAEL013245 | CLIPB28 |
| AAEL000087 | TEP22 |
| AAEL003723 | LYSC11 |
| AAEL001435 | SPZ2 |
| AAEL008596 | SPZ3A |
