# Supplementary material for: A Critical Role for CLSP2 in the Modulation of Antifungal Immune Response in Mosquitoes
Source: PLoS Pathog. 2015 Jun 9;11(6):e1004931. doi: 10.1371/journal.ppat.1004931 (PMC4461313; doi:10.1371/journal.ppat.1004931)
Supplement: S6 Table — (DOCX) [file ppat.1004931.s011.docx]

| Table S6 Primers used for Real-time RT-PCR, RT-PCR, dsRNA synthesis, and protein expression | | | | |
| --- | --- | --- | --- | --- |
| Primers for Real-time RT-PCR | | | | |
| PPO1 | TCAGTGACGATGCAGAGACC | | | |
|  | AGCAATCTTGCGATGCTTTT | | | |
| PPO2 | GTCGTTCGGAGCAATCTAGC | | | |
|  | GTACCCTTGGGAATCAGCAA | | | |
| PPO3 | TTGCTAAGAAAGATCGCCGT | | | |
|  | AATCGGTGCCTGTAGATTGG | | | |
| PPO4 | GAACGGTTTTGCAATGGTCT | | | |
|  | AGTTGATTTCGACCACGACC | | | |
| PPO5 | ATGACCGACTGGAAAACTGG | | | |
|  | ACGTTTGGCCTCCATACTTG | | | |
| PPO6 | TTCTGCAATCGCTTACAACG | | | |
|  | CAGAGAGTCCAATTTGGGGA | | | |
| PPO7 | TAATAAGGATCGCCGAGGTG | | | |
|  | TACGGTTCTCGGATTGGTTC | | | |
| PPO8 | ACGTCCTGTTGACCTTTTGG | | | |
|  | GGGTGCATGTTGAATGTGAG | | | |
| PPO9 | TCGAATCACTCGTCGATCTG | | | |
|  | TATCCGGAAGATCCTTGTCG | | | |
| PPO10 | ATTATGGCGTGATGGGTGAT | | | |
|  | TACGGAGTGAGCGAGTCCTT | | | |
| SRPN1 | GAATCGAACCTACAACGGGA |  |  |  |
|  | CGGTAGCTCAGCTTTATCGG | | | |
| SRPN2 | AACGCAGTGATATCGCCTCT | | | |
|  | AGATCACCGTTTGGTTCCAG | | | |
| DefA | TAGTGCTTGTGCTGCTCATTG | | | |
|  | TAGGTATAAGTTGCTCGAAGG | | | |
| CecA | TGGCTGTTCTTCTCCTGACC | | | |
|  | CCCCAGCTACAACAGGAAGA | | | |
| CLIPB9 | CCAGTGGAAGAAAAGCTTGC | | | |
|  | TTCTTGAAATTGGACGAGGG | | | |
| CLIPB39 | CACGCAAGCTGATAGCACAT | | | |
|  | AGTCCGAATACGTCGGAATG | | | |
| CLIPB79 | ATCGGTACGGCTAGGAGAAA | | | |
|  | GATGTCCTGTGGAGGAGGTG | | | |
| CecE | GTTCTGCTCATCGGGTTGGT | | | |
|  | GTTACTACGGGAAGTGCTTT | | | |
| FREP3 | AGGAAGCCATCAACCAGAAT | | | |
|  | GTTCGCAGTAGGCCAGAAAA | | | |
| FREP5 | GTGGTATCCCAGATGCTATT | | | |
|  | ATCTTTGAAACACGCAGTCC | | | |
| FREP10 | AAGGCAGAGCCGAGTAAGAC | | | |
|  | TGTAGAACTCCACCGAACCA | | | |
| Domeless | AGGAATGGAATCCCAATCAG | | | |
|  | ATCTCCGAGCAGGAATAAAA | | | |
| JAK | CGAGGATGGCGTCAGCGTAA | | | |
|  | GGCCGGGTTGAAGAACAGAA | | | |
| STAT | CAGCATCGTCGGCTTCATCC | | | |
|  | TTGACCCAGGCGATTGTGAT | | | |
| TEP2 | CTGGCGAGAAGACCATCACT | | | |
|  | CGAAGAACGGTAGGAAGACG | | | |
| CLIPB29 | ATCTGGCATTGTTCCGTCTG | | | |
|  | ACATTGGTTTGTCGGCGTTA | | | |
| PGRP-LC | AACTTTCTGATCGGAGGTGA | | | |
|  | ACGCTATGCCAATACTGTCG | | | |
| GNBP1 | CACCAATGCTATTCCCAACT | | | |
|  | CGATGTCCAATCAGCCAGTA | | | |
| CLSP1 | TGCGTGGTAATCACCCTGTT | | | |
|  | TGAAGTTGAACGACGAAAGC | | | |
| CLSP2 | GTTGGGGTGGTGTAAAATGG | | | |
|  | ACGACCAGTGTGGACTGACA | | | |
| Toll5B | TGCTGGCTAATCTGGACCTT | | | |
|  | CATTTCTGGCAGTGTTTCGA | | | |
| IKK1 | ATTACGGCTTGATGCTGAAC | | | |
|  | ATTGACCTTCGCCATCTCCT | | | |
| Rel1 | GTTCCTTCGTTTCAACCCTC | | | |
|  | TGGACCATTAGCGATTTCAG | | | |
| Rel2 | TTTGAATGTGCTGTTGGGTC | | | |
|  | GAATGTTGTTTCCGTGCTTA | | | |
| Cactus | GCAGATGAAGTCCAAGGAGC | | | |
|  | GATCACGGCAAGGTGTAGGT | | | |
| RPS7 | TCAGTGTACAAGAAGCTGACCGGA | | | |
|  | TTCCGCGCGCGCTCACTTATTAGATT | | | |
| CLIPB8 | GATGACGAAGTCTGTGGGTA | | | |
|  | GCATCCGTGAACAATCTTTT | | | |
| CLIPB16 | ATCGTTCAAGGCGACTACTA | | | |
|  | CCAGAGCAAGGGTAAATAAA | | | |
| CLIPB24 | AAGGTGCAGAATGAAGTGAA | | | |
|  | TAGTGGCAAGCAAATAGGAC | | | |
| CLIPB28 | CGTATGTTGCGATGAGACAG | | | |
|  | TATCGTCCAGGTTGGTTCTA | | | |
| CLIPB13B | AGGAAGTAGATTGCGAGGTA | | | |
|  | CGATTGTAGTCGCTATGAAA | | | |
| CLIPB46 | GCGGTAGTAGATGCTTTGTG | | | |
|  | ACGGTGCTGTTCAGCTTTAT | | | |
| HPX3 | TCCTCACCCACCATACTGCG | | | |
|  | TTGTTGGAAAGCGTTCGTCT | | | |
| TEP22 | TACCTCCTGGTACGTGACCG | | | |
|  | TACTTCACCTCGCTTTATCG | | | |
| LYSC11 | AAAGCTCTACTCGCTCAAGG | | | |
|  | TTGCGATTGTTGTTCTTCTT | | | |
| SPZ2 | TCGTTTGCTCAGTTGGGACA | | | |
|  | CGTTGAATGGCGGGAAAGTC | | | |
| SPZ3A | CCCTTCCCAAACCTATCCTC | | | |
|  | GAACCCTCGCCATACGTTCT | | | |
| CECF | TGGTCGCCCTAGTCTTGCTG | | | |
|  | CACTCGCTTGCCGACTCCTT | | | |
| Primers for RT-PCR | | | | |
| Actin | AAGGCCAACCGTGAGAAGATGACT | | | |
|  | GCTCGTTGCCAATGGTGATGAC | | | |
| CLSP1 | CTTCCAGTGTGGAATACGGC | | | |
|  | TTACGACCAGTTTAGACTAA | | | |
| CLSP2 | TGGAATAAGGCAATACAAAAC | | | |
|  | TTACGACCAGTGTGGACTGA | | | |
| Primers for dsRNA synthesis | | | | |
| iCLSP2 | T7-ATACCACGGACCATTCGTGT | | | |
|  | T7-GTACAAGGACAAGGCGAAGC | | | |
| CLIPB24 | T7-AAGGTGCAGAATGAAGTGAA | | |  |
|  | T7-TAGTGGCAAGCAAATAGGAC | | |  |
| CLIPB28 | T7-CGCCAAACGAGCCGTAACTC | | |  |
|  | T7-GTTCACCCGCAGCAGTCCAA | | |  |
| CLIPB13B | T7-CACGCTCGTATTTACCTATC | | |  |
|  | T7-CACATCTGTTTACTGCCAAG | | |  |
| CLIPB46 | T7-GGACGGCAATGCTCGCTTAT | | |  |
|  | T7-CAACGGACCACCGGAATCAC | | |  |
| HPX3 | T7-CTCCTCACCCACCATACTGC | | |  |
|  | T7-TCACGTCTATTCCTGCCAAT | | |  |
| TEP22 | T7-GAGAATCGGCTCGACCTTAT | | |  |
|  | T7-TACCTGGTTGTGCGGTGCTA | | |  |
| LYSC11 | T7-TTGATTGTACTGGTGGGTCT | | |  |
|  | T7-AAGCCTTACTGATGTCGTCT | | |  |
| SPZ2 | T7-GTTTGCTCAGTTGGGACACG | | |  |
|  | T7-AGACGATTTCTGGACGGTTG | | |  |
| SPZ3A | T7-CAGATCCCAACGATCAAGAC | | |  |
|  | T7-GCATTCACCTTCACAAACCC | | |  |
| Rel1 | T7-TTCTTGACGCACTGGATGC | | |  |
|  | T7-GGACCAAACGACAACAACG | | |  |
| Primers for protein expression | | | | |
| PPO1 | ATGCGAGCTCTCTAGAGGGTATTAATAATGGCATCAGGAAGT | | | |
|  | ATGCCTCGAGTCAGTGGTGGTGGTGGTGGTGTGTATTAGTAAAGCG | | | |
| PPO3 | ATGCGAGCTCTCTAGAGGGTATTAATAATGGCTGAGAGTAAA | | | |
|  | ATGCCTCGAGTCAGTGGTGGTGGTGGTGGTGGCTTATAATCGTATT | | | |
| PPO5 | ATGCGGATCCTCTAGAGGGTATTAATAATGACCGACTGGAAA | | | |
|  | ATGCGAGCTCTCAGTGGTGGTGGTGGTGGTGTTTGGAAATGGTGGT | | | |
| PPO8 | ATGCGGATCCTCTAGAGGGTATTAATAATGGCATCAAACAGC | | | |
|  | ATGCGAGCTCTCAGTGGTGGTGGTGGTGGTGTGTATTGGTGAAGCG | | | |
| rlectin (CLSP2) | GGATGACGACGATAAGATGTGCTTACATCAGCCAACAA | | | |
|  | AGATGAGCTTCTGCTCCGACCAGTGTGGACTGACA | | | |
|  | ATGC GGATCCTGCTTACATCAGCCAACAA | | | |
|  | ATGCGAGCTCCGACCAGTGTGGACTGACA | | | |
| CLSP1 | ATGCGGATCCGGAATACGGCAAGACAAA | | | |
|  | ATGCGAGCTCAACAGATTCACAAATGTAGCG | | | |
| CLSP2 | ATGCGGATCCGGAATAAGGCAATACAAAAC | | | |
|  | ATGCGAGCTCCGACCAGTGTGGACTGA | | | |
