# Supplementary material for: A Critical Role for CLSP2 in the Modulation of Antifungal Immune Response in Mosquitoes
Source: PLoS Pathog. 2015 Jun 9;11(6):e1004931. doi: 10.1371/journal.ppat.1004931 (PMC4461313; doi:10.1371/journal.ppat.1004931)
Supplement: S7 Table — (DOCX) [file ppat.1004931.s012.docx]

Table S7. Repertoire of accession numbers/ID numbers for genes and proteins involved in the text.

| Name | ID/Accession numbers |  | Name | ID/Accession numbers |  | Name | ID/Accession numbers |
| --- | --- | --- | --- | --- | --- | --- | --- |
| CLSP2 | AAEL011616 |  | SPZ3A | AAEL008596 |  | Dm GNBP3 | NM_079262 |
| TEP22 | AAEL000087 |  | Toll5B | AAEL003507 |  | Ms HP14 | AY380790 |
| Spz1C | AAEL013433 |  | MyD88 | AAEL007768 |  | Ag CTL4 | XP_315348 |
| Toll5A | AAEL007619 |  | Tube | AAEL007642 |  | Ag TLMA2 | XP_315347 |
| CLIPB5 | AAEL005064 |  | Pelle | AAEL006571 |  | Bm TEP1 | XP_004927010 |
| CLIPB29 | AAEL006674 |  | TARF6 | AAEL011363 |  | Bm TEP2 | XP_004927013 |
| PPO1 | AAEL013498 |  | Cactus | AAEL000709 |  | Bm TEP3 | XP_004927018 |
| PPO2 | AAEL013499 |  | Rel1 | AAEL007696 |  | Tc TEP1 | EFA04694 |
| PPO3 | AAEL011763 |  | TEP2 | AAEL014755 |  | Tc TEP2 | EFA07508 |
| PPO4 | AAEL013501 |  | FREP5 | AAEL009384 |  | Tc TEP3 | XP_970922 |
| PPO5 | AAEL013492 |  | FREP10 | AAEL008646 |  | Tc TEP4 | EEZ99236 |
| PPO6 | AAEL014544 |  | PGRP-LC | AAEL014640 |  | Am TEP7 | XP_006565503 |
| PPO7 | AAEL013493 |  | IMD | AAEL010083 |  | Am TEPA | XP_397416 |
| PPO8 | AAEL013496 |  | FADD | AAEL001932 |  | Am TEPB | XP_00662096 |
| PPO9 | AAEL014837 |  | Dredd | AAEL007642 |  | Dm Tep4 | NP_523603 |
| PPO10 | AAEL011764 |  | TAK1 | AAEL007035 |  | Dm Tep3 | NP_523507 |
| CLSP1 | AAEL011622 |  | IKK1 | AAEL003245 |  | Dm Tep5 | NP_609988 |
| DEFA | AAEL003841 |  | Rel2 | AAEL007624 |  | Dm Tep6 | NP_524688 |
| CECA | AAEL000627 |  | Domeless | AAEL012471 |  | Flyb TEP4 | NP_523578 |
| CECE | AAEL000611 |  | JAK | AAEL012553 |  | Dm Tep2A | NP_523506 |
| CECF | AAEL000625 |  | STAT | AAEL009692 |  | Ag TEP13 | XP_317044 |
| GNBP1 | AAEL003889 |  | TEP20 | AAEL001794 |  | Ag TEP3 | XP_315149 |
| SRPN1 | AAEL014079 |  | TEP21 | AAEL001802 |  | Ag TEP11 | XP_318490.2 |
| SRPN2 | AAEL014078 |  | TEP23 | AAEL001163 |  | Ag TEP12 | XP_314753 |
| CLIPB9 | AAEL003610 |  | TEP24 | AAEL017023 |  | Ag TEP6 | XP_318490.1 |
| CLIPB39 | AAEL003632 |  | TEP3 | AAEL008607 |  | Ag TEP2 | XP_555086 |
| CLIPB79 | AAEL014139 |  | TEP13 | AAEL012267 |  | Ag TEP15 | XP_317088 |
| SPZ2 | AAEL001435 |  |  |  |  | Ag TEP9 | XP_309879 |
|  |  |  |  |  |  | Ag TEP4 | XP_318493 |
|  |  |  |  |  |  | Ag TEP1 | CBA02654 |
|  |  |  |  |  |  | Ag TEP10 | XP_309892 |
|  |  |  |  |  |  | Ag TEP14 | XP_317085 |
